# Effects of Melatonin on Anterior Pituitary Plasticity: A Comparison Between Mammals and Teleosts

**DOI:** 10.3389/fendo.2020.605111

**Published:** 2021-01-11

**Authors:** Elia Ciani, Trude M. Haug, Gersende Maugars, Finn-Arne Weltzien, Jack Falcón, Romain Fontaine

**Affiliations:** ^1^Department of Pharmacy, Faculty of Mathematics and Natural Sciences, University of Oslo, Oslo, Norway; ^2^Department of Oral Biology, Faculty of Dentistry, University of Oslo, Oslo, Norway; ^3^Physiology Unit, Faculty of Veterinary Medicine, Norwegian University of Life Sciences, Oslo, Norway; ^4^Laboratoire Biologie des Organismes et Ecosystèmes Aquatiques (BOREA), MNHN, CNRS FRE 2030, SU, IRD 207, UCN, UA, Paris, France

**Keywords:** melatonin, adenohypophysis, photoperiod, melatonin receptors, seasonal reproduction, plasticity, endocrinology, light

## Abstract

Melatonin is a key hormone involved in the photoperiodic signaling pathway. In both teleosts and mammals, melatonin produced in the pineal gland at night is released into the blood and cerebrospinal fluid, providing rhythmic information to the whole organism. Melatonin acts *via* specific receptors, allowing the synchronization of daily and annual physiological rhythms to environmental conditions. The pituitary gland, which produces several hormones involved in a variety of physiological processes such as growth, metabolism, stress and reproduction, is an important target of melatonin. Melatonin modulates pituitary cellular activities, adjusting the synthesis and release of the different pituitary hormones to the functional demands, which changes during the day, seasons and life stages. It is, however, not always clear whether melatonin acts directly or indirectly on the pituitary. Indeed, melatonin also acts both upstream, on brain centers that control the pituitary hormone production and release, as well as downstream, on the tissues targeted by the pituitary hormones, which provide positive and negative feedback to the pituitary gland. In this review, we describe the known pathways through which melatonin modulates anterior pituitary hormonal production, distinguishing indirect effects mediated by brain centers from direct effects on the anterior pituitary. We also highlight similarities and differences between teleosts and mammals, drawing attention to knowledge gaps, and suggesting aims for future research.

## Introduction

Our environment is constantly changing. While some variations are fast and unpredictable (e.g. meteorological phenomena), others, such as solar cycles, moon phases, and seasons follow regular patterns. Photoperiod, the alternation of light and darkness, is the most reliable (noise-free, characterized by predictable rhythms over a long period of time) signal, allowing animals to synchronize their biological rhythms with both daily and seasonal changes. Photoperiod is conveyed by two types of signal: a neural message from photoreceptive structures to specific signaling centers in the brain, and a hormonal message (1, 2).

**Figure 1:**
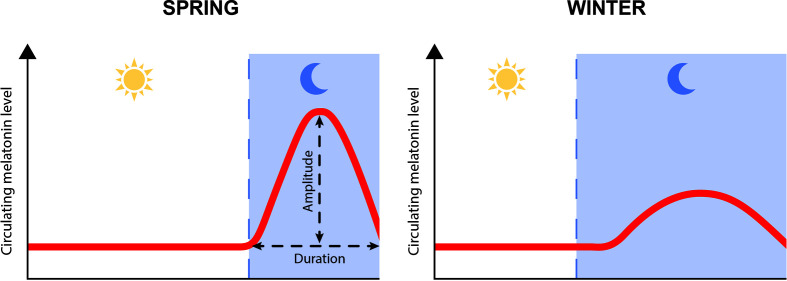
Schematic representation of daily and seasonal fluctuation in plasma melatonin levels.

Melatonin is the key hormone that conveys rhythmic information from the environment, including photoperiod and temperature, to the organism. Circulating blood levels of melatonin exhibit a daily rhythm with higher levels during night than during day, and a seasonal rhythm with longer duration of the high level period during winter, as a consequence of the longer dark phase (**Figure 1**). Additionally, variations in temperature fine-tune those rhythms by modulating the amplitude of melatonin production. Duration and amplitude of melatonin release therefore provide clear information regarding time of the day and the year, and allow the synchronization of metabolic, physiological, and behavioral events, including growth, reproduction, and migration (3, 4).

Melatonin is synthesized from tryptophan in four enzymatic steps (4, 5). Tryptophan is first converted into 5-hydroxytryptophan by the tryptophan hydroxylase, then converted into serotonin by the 5-hydroxy-tryptophan decarboxylase. Afterwards, serotonin is acetylated by the arylalkylamine *N*-acetyltransferase (AANAT), producing *N*-acetylserotonin, which is finally converted into melatonin by the hydroxyindole-*O*-methyl transferase. AANAT has been reported to be the limiting enzyme driving the rhythm of melatonin production (6). It has been hypothesized that the functional shift of AANAT from amine detoxification to melatonin synthesis played a critical role in the evolution of melatonin as a night-time signal (7–9).

While the general mechanism of melatonin synthesis is conserved across vertebrates, the number of genes encoding the different enzymes differs between mammals and fish, as a consequence of whole genome duplications that occurred in the vertebrate lineage. Indeed, after the two successive whole genome duplications (referred to as 1R and 2R) which occurred at the base of the vertebrate lineage (10–12), a third one (3R) occurred at the base of the teleost fish lineage (13), and a fourth one (4R) occurred independently in both the cyprinid and salmonid lineages (14, 15). Following a genome duplication, one of the paralogous genes may be lost or duplicated paralogues may acquire differential specialized functions over time, and an increase in the number of paralogues, expands the hormone-receptor combinations (16). In contrast to mammals, all actinopterygians, including the teleosts, possess at least two *aanat* genes (*aanat1* and *aanat2*) (9, 17), resulting probably from the whole genomic duplications that occurred in the vertebrate lineage (18). Additionally, *aanat1* and *aanat2* have also been duplicated during the 3R (18). While one of the *aanat2* paralogues was lost early after the 3R, this was not the case for the *aanat1* paralogues and, to date, some fish possess two Aanat1 isoforms (*aanat1a* and *aanat1b*) or either one of them. While *aanat1* genes are mostly expressed in the retina, brain, and peripheral tissues, *aanat2* expression is specific to the pineal gland (19, 20), the site of production of circulating melatonin in both mammals (21) and teleosts (22, 23). Melatonin is then released from the pineal gland into the blood and cerebrospinal fluid to be transported to its target organs.

Melatonin acts through several different receptors (MTNR), belonging to the G‐protein coupled receptor superfamily (24). Four sub‐groups of Mtnr, arising from the 1R and 2R, have been characterized in vertebrates: MTNR1A (Mel1a or MT1), MTNR1B (Mel1b or MT2), MTNR1C (Mel1c or GPR50), and MTNR1D (Mtnr1A-like or Mel1d) (25–28). In mammals, melatonin action is mediated only through two MTNR paralogues, MTNR1A and MTNR1B, since the *Mtnr1d* gene was lost in the mammalian lineage and MTNR1C lost its ability to bind melatonin (28). Teleosts may possess up to 7 Mtnr paralogues (excluding the polyploid cyprinids), arising from the 3R and 4R (25, 28). MTNR affects different intracellular signaling pathways, including cAMP/PKA, *via* G_i_ proteins (MTNR1A and MTNR1B) (29, 30), PLC/PKC *via* G_q_‐proteins (MTNR1A and MTNR1C) (31) and cGMP *via* G_i/o_ proteins (MTNR1B) (32, 33). In medaka (*Oryzias latipes*), all four Mtnr subtypes are functional and decrease cAMP in response to melatonin exposure (27). Interestingly, melatonin receptors in Atlantic salmon (*Salmo salar*) increase cAMP when activated by melatonin (25). The broad distribution of MTNR expression in the central nervous system (including the pituitary) and peripheral tissues suggests melatonin can have widespread effects (28, 34).

**Figure 2:**
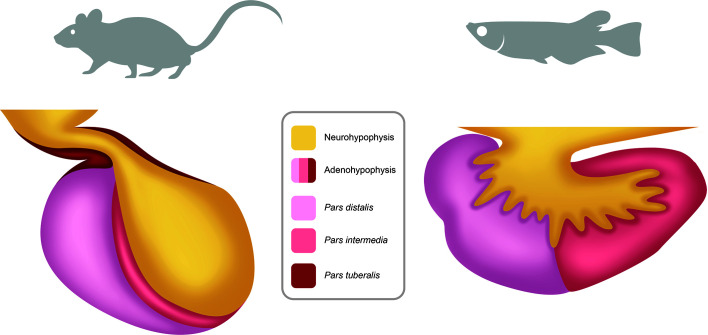
Schema of the pituitary in mammals and teleosts. The pituitary is composed of two main parts: the neurohypophysis (posterior pituitary) and the adenohypophysis (anterior pituitary). The neurohypophysis is mainly composed of neuron terminals from neuroendocrine cells with cell soma located in the preoptic-hypothalamic region of the brain. The adenohypophysis contains different hormones producing cell types and can be anatomically divided in *pars distalis*, *pars intermedia* and, in mammals but not in teleosts, *pars tuberalis*.

The pituitary is a key endocrine gland in all vertebrates, involved in the regulation of many important physiological processes (35). These include growth, puberty, seasonal sexual maturation, metabolism, and homeostasis, which exhibit cycling components over the day, the year and the life cycle. Located below the hypothalamus, the pituitary is composed of two main parts with different developmental origins (36): the anterior pituitary (adenohypophysis) and the posterior pituitary (neurohypophysis) (**Figure 2**). The neurohypophysis originates from a down-growth of the diencephalon and is mainly composed of nerve terminals from neuroendocrine cells in the preoptic area (POA) and the hypothalamus of the brain, which are considered today as two distinct regions (37). The adenohypophysis originates from an up-growth of the pharyngeal ectoderm and endoderm (38) and can be histologically divided in the *pars intermedia* (PI), the *pars distalis* (PD), and the *pars tuberalis* (PT), the latter present in mammals but not in teleosts. The adenohypophysis hosts several hormone-producing cell types: gonadotropes (producing the gonadotropins: follicle-stimulating and luteinizing hormones, FSH and LH), lactotropes (prolactin, PRL), somatotropes (growth hormone, GH), thyrotropes (thyrotropin, TSH), corticotropes (adrenocorticotropin, ACTH), and melanotropes (melanocyte-stimulating hormone, MSH) (39). Teleosts also possess one additional cell type, the somatolactotropes responsible for the production of somatolactin (Sl) (40).

The activity of pituitary endocrine cells is constantly changing over time, adjusting the hormonal production to changing physiological needs. It is controlled by factors produced from signaling centers in the brain, mainly the POA and hypothalamus, and from peripheral organs, which provide positive and negative feedback to these centers and to the pituitary (41, 42). In mammals, POA/hypothalamic neurons project to the median eminence (ME) of the hypothalamus, releasing their hormones into the hypophysial portal system where they are transported *via* the blood stream to the pituitary endocrine cells (42). Teleosts, on the other hand, lack the hypophysial portal system, and instead the POA/hypothalamic neurons innervate the pituitary, releasing their neurohormones directly at target cells or into pituitary blood vessels (41, 43). Pituitary hormonal production is regulated through both modulation of the activity of individual cells, and regional reorganizations of the anterior pituitary in terms of structure or cell composition, as discussed previously for gonadotropes (38, 44).

While pituitary plasticity is influenced by environmental factors, the role that melatonin plays in translating fluctuations of environmental conditions into pituitary hormonal production is not always clear. In addition, the mechanisms of melatonin action are complex, as both direct effects on pituitary endocrine cells and indirect effects through neuro/hormonal signaling centers combine to regulate pituitary activity. In this review, we describe the known pathways through which melatonin modulates anterior pituitary hormonal production, distinguishing between indirect effects mediated by brain centers and direct effects on the anterior pituitary. We also highlight similar and divergent features between teleosts and mammals, and emphasize important unsolved questions for future research.

## Brain-Mediated Effects of Melatonin on Anterior Pituitary Endocrine Cells

### Mammals

**Figure 3:**
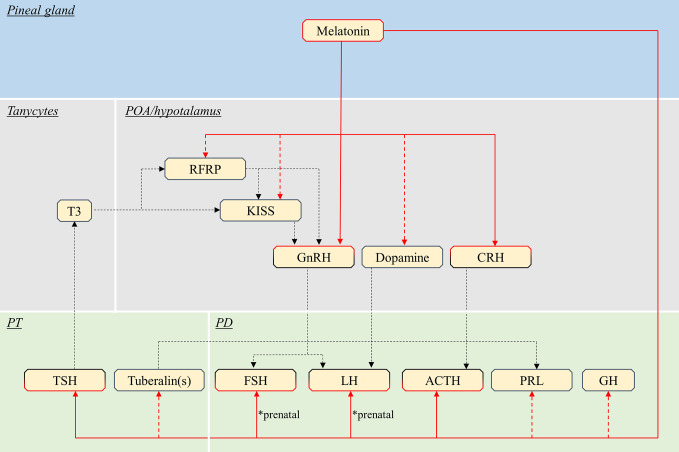
Schematic view of the putative pathways through which melatonin influence pituitary endocrine activity in mammals. Red continuous lines indicate cell types directly targeted from melatonin. Dashed red lines indicate cells influenced by melatonin *via* yet unidentified interneurons, paracrine signals or MTNR. Note that melatonin might act only on a few of the illustrated pathway, in different species (see text). Black dashed lines indicate all other interactions between brain and pituitary. POA, preoptic area; PT, *pars tuberalis*; PD, *pars distalis*; T3, triiodothyronine; RFRP, RFamide-related peptide; KISS, kisspeptin; GnRH, gonadotropin-releasing hormone; CRH, corticotropin-releasing hormone; TSH, thyroid-stimulating hormone; FSH, follicle-stimulating hormone; LH, luteinising hormone; ACTH, adrenocorticotropic hormone; PRL, prolactin; GH, growth hormone.

Endocrine pituitary cells are primarily controlled by brain signaling centers, mainly the preoptic and hypothalamic area (42) (**Figure 3**, **Table 1**), which integrate nervous and hormonal signals of different origins. These brain regions are characterized by the presence of numerous melatonin binding sites as shown in rodents and ruminants (60–66). Although the suprachiasmatic nucleus (SCN) of the hypothalamus drives the rhythmic production of melatonin in mammals (67), the present review will focus on known effects of melatonin on brain centers directly regulating pituitary endocrine production, as discussed below.

**Table 1:** Summary of the known effects of melatonin POA/hypothalamic neurons controlling pituitary hormonal production in mammals.

Target	Effect of melatonin	Species	Breeding season/Photoperiod	Description	Reference
**Mammals**					
GnRH	Stimulates	Sheep	Winter/SP	Melatonin administration increases GnRH secretion	Bittman et al. (45) Viguié et al. (46)
	Inhibits	Jerboa	Summer/LP	Short photoperiod and melatonin administration downregulate GnRH release	El Qandil et al. (47)
	Inhibits	GT1-7 mouse GnRH cell line		Melatonin reduces GnRH mRNA and protein levels in GT1-7 cell line	Roy et al. (48)
	Modulates	Rat	non-seasonal breeder	Melatonin augments/reduces GABA-induced currents in GnRH neurons in a sex dependent way	Sato et al. (49)
KISS	Inhibits	Syrian hamster	Summer/LP	Melatonin reduces KISS1 mRNA	Revel et al. (50) Ansel et al. (51)
		Turkish hamster	Summer/LP	Melatonin reduces KISS1 mRNA	Piekarski et al. (52)
		Striped hamster	Summer/LP	Melatonin reduces KISS1 mRNA	Li et al. (53)
		Rat	non-seasonal breeder	Melatonin reduces KISS1 mRNA	Oliveira et al. (54)
RFRP	Inhibits	Syrian hamster	Summer/LP	Melatonin (and SP) reduces RFRP-3 mRNA and protein	Mason et al. (55) Revel et al. (56)
		Siberian hamster	Summer/LP	Melatonin (and SP) reduces RFRP-3 mRNA and protein	Ubuka et al. (57) Revel et al. (56)
Dopamine	Stimulates	Syrian hamster	Summer/LP	Melatonin administration stimulates tyrosine hydroxylase activity in males	Alexiuk et al. (58)
	Inhibits	Sheep	Winter/SP	Melatonin implants inhibit tyrosine hydroxylase activity	Viguié et al. (59)

#### Gonadotropes

Gonadotropes are the most investigated pituitary cell type in relation to melatonin, due to the high scientific and economic interest around the seasonal control of reproduction. Indeed, gonadotropes produce the two gonadotropins (FSH and LH), key hormones in the control of reproduction, which are heterodimeric glycoproteins composed of a common α-subunit (GPHα, also shared with TSH) and a hormone-specific β subunit (LHβ or FSHβ) conferring the specific biological activity (42).

##### GnRH

Mammalian gonadotropin-releasing hormone (GnRH1 or mGnRH-I), a 10-amino acid neuropeptide produced from POA/hypothalamic neurons, is the main regulator of gonadotropin synthesis and secretion (42). Most mammals also possess a second form (GnRH2 or cGnRH-II), expressed in the midbrain and other organs, which is primarily involved in other functions than regulating gonadotropin release. Vertebrates also possess two major types of GnRH receptors (type I with the GnRHR1a and II with the GnRHR2c) (68), however in many mammalian species, GnRHR2c receptor is not functional (69).

Melatonin influences GnRH production and thus the reproductive axis in seasonal breeders. Melatonin administration and short photoperiod (SP) cause testicular regression in the male summer breeder jerboa (*Jaculus orientalis*), a desert hibernating rodent, by inhibiting GnRH release (47). In contrast, melatonin administration in the ewe (*Ovis aries*, a winter breeder) increases the pulsatile GnRH secretion from hypothalamus, and pituitary LH secretion (45, 46). While suggesting a connection between melatonin level and GnRH production, these *in vivo* experiments do not reveal whether melatonin acts directly on GnRH neurons, indirectly *via* interneurons or through a combination of both. However, *in vitro* experiments using the GT1-7 mouse hypothalamic GnRH cell line reveal that GnRH neurons express MTNR1A (MT1) and MTNR1B (MT2) (48) and demonstrate that melatonin inhibits both GnRH mRNA expression and protein secretion (48, 70).

Melatonin action might be modulated in a sexually dimorphic way in rodents as higher mRNA levels of *Mtnr1a* (MT1) are detected in male than female rat (*Rattus norvegicus*) GnRH neurons, while *Mtnr1b* (MT2) is not expressed in either sex (71). Sexual dimorphism of the melatonin response in GnRH neurons is supported by another *in vitro* study, where melatonin augmented the membrane current induced by gamma-aminobutyric acid (GABA_A_) in 70% and attenuated it in 18% of neurons from adult males, while it augmented the current in only 25% and attenuated it in 61% of the neurons from adult females (49). Nevertheless, the physiological relevance of the direct actions of melatonin on GnRH neuron activity *in vivo* remains controversial, as melatonin might additionally act on upstream signals, such as KISS1, RFRP3 and T3, as discussed below.

##### RFRP3 (GnIH)

RFamide related peptide3 (RFRP3) is the mammalian orthologue of avian GnIH, which was originally identified in birds as an inhibitory factor of gonadotropin synthesis and release, by acting on both GnRH neurons and gonadotropes. RFRP3 neurons are located in the paraventricular nucleus of the hypothalamus (42). Interestingly, the effects of RFRP3 on gonadotropin synthesis are deeply influenced by sex and timing of administration in mammals. For instance, in Syrian hamsters (*Mesocricetus auratus*), RFRP3 inhibits gonadotropin secretion in ovariectomized females (72) while it stimulates GnRH and gonadotropin secretion in males (73). In male Siberian hamster (*Phodopus sungorus*), RFRP3 directly injected into the third ventricle inhibits LH release when applied under LP, but has excitatory effects under SP (57), suggesting that melatonin might differentially influence the activity of RFRP3 neurons over the seasons.

In summer breeders, such as Siberian and Syrian hamster, both SP and melatonin injection reduces RFRP3 protein and mRNA levels, as well as decrease RFRP3 fibre density and number of projections to GnRH neurons (55–57). These studies also show that pinealectomy abolishes the effects of photoperiod manipulation, while subsequent melatonin exposure re-establishes them. While sex steroids are known to induce positive or negative feedback on hypothalamic signaling centers, the SP-induced reduction in RFRP3 protein and mRNA levels observed in male hamsters is not a consequence of reduced circulating steroid levels, since neither castration nor testosterone implants alter RFRP3 synthesis. These data therefore strongly suggest that melatonin is responsible for the inhibition of RFRP3.

In winter breeders, such as sheep, SP decreases both RFRP3 mRNA and protein levels, and RFRP3 neuron projections to GnRH neurons (74, 75). Similarly, in brushtail possum (*Trichosurus vulpecula*) females, the number of RFRP3 neurons decreases during winter (76). Interestingly, in the laboratory Wistar rat, a non-photoperiodic breeder, no effect of photoperiod manipulation was detected on RFRP3 neurons (56). These results suggest that the photoperiodic control of melatonin on RFRP3 is conserved among mammals, with inhibiting effects in both summer and winter breeders, while the downstream effects of the RFRP3 system on gonadotropin secretion might diverge to adapt to long-day or short-day breeding strategies.

Whether melatonin acts directly on RFRP cells in mammals requires further investigations as there is still a lack of evidence for colocalization with MTNR, or studies clearly demonstrating a direct action of melatonin on RFRP3 neurons, as previously discussed by Kriegsfeld and collaborators (77).

##### Kisspeptin

KISS neurons produce the neuropeptide kisspeptin (KISS) and stimulate GnRH synthesis and secretion, thereby regulating gonadotrope cell activity (78). Located in two discrete hypothalamic nuclei, the arcuate nucleus (ARC) in all mammals and the anteroventral periventricular area around the 3^rd^ ventricle in rodents or the POA in non-rodent mammals, the activity and number of KISS neurons display a marked photoperiodic/seasonal pattern, as shown below.

In the winter breeding sheep, SP upregulates both ARC *KISS1* mRNA and protein, and increases the number of both ARC KISS neurons and synaptic connections from KISS to GnRH neurons (75). In contrast, melatonin inhibits the activity of KISS neurons in summer breeders. For instance, using a combination of photoperiod manipulation, pinealectomy and melatonin administration Revel and colleagues (50) and Ansel and colleagues (51) demonstrated that melatonin clearly reduces ARC *KISS1* mRNA in Syrian hamsters, an effect further modulated by the negative steroid feedback. Similar inhibitory effects of melatonin on *KISS1* mRNA were detected in Turkish (*Mesocricetus brandti*) (52) and striped (*Cricetulus barabensis*) (53) hamsters but also in the rat, a non-seasonal breeder (54). Interestingly, in the Siberian hamster, ARC *KISS1* mRNA levels are lower under LP due to a robust negative sex steroid feedback overriding the melatonin signal, since castration in LP animals restores high *KISS1* mRNA levels (79). Therefore, the role of melatonin among different species, or different reproductive stages, might be difficult to identify considering the impact of steroid feedbacks on ARC KISS neurons.

Although a direct effect of melatonin on *KISS1* mRNA levels was detected in a hypothalamic cell line from rat (80), *Mtnr* expression has not been found in sheep KISS neurons, neither during the breeding nor during the non-breeding season (81). These results suggest that the effects of melatonin on KISS neurons could be mediated upstream.

##### Dopamine

The activity of the dopaminergic neurons located within the POA/hypothalamic area, which are involved in the inhibition of gonadotropin synthesis and release (82), also appears to be regulated by melatonin. For instance, in the ewe, a winter breeder, melatonin implants inhibit the activity of tyrosine hydroxylase (the rate-limiting enzyme in the dopamine synthesis pathway) in the median eminence, while stimulating LH release (59). In contrast, in male Syrian hamsters, in which SP elicits testicular regression, melatonin administration stimulates tyrosine hydroxylase activity in the median eminence, increasing dopamine synthesis and release (58).

#### Other Endocrine Cells in the Pars Distalis

Melatonin plays a role in the regulation of other pituitary endocrine cell types in mammals by regulating their main hypophysiotropic factors.

Corticotrope cells produce ACTH, a hormone involved in various physiological processes including the stress response (promoting the release of cortisol from the adrenal gland) and the control of numerous daily and seasonal physiological rhythms (including sleep) (83). Corticotropes are mainly regulated by corticotropin releasing hormone (CRH) neurons located in the paraventricular nucleus. Melatonin exerts a stress-protective effect in mammals (84, 85). Daily melatonin administration reduces the ACTH secretory response to acute and chronic stress in rat (86, 87). In humans (*Homo sapiens*), oral melatonin administration in blind individuals normalizes the temporal pattern of ACTH and cortisol plasma concentrations during sleep, suppressing the pituitary-adrenal activity during early sleep and activating it during late sleep (88). Melatonin might modulate ACTH production by acting directly on hypothalamic CRH neurons, which express the MTNR1A in humans (89).

Lactotrope cells produce PRL, a peptide hormone involved in reproduction (lactation), moulting, metabolism, and immune responses. PRL secretion is stimulated by releasing factors from the PT and inhibited by dopamine secreted by tubero-infindibular dopaminergic neurons located in the dorsomedial arcuate nucleus (90–92). Exogenous melatonin administration and SP decrease the PRL secretion in ruminants. For instance, oral melatonin administration inhibits PRL secretion in lactating ewes (93). SP reduces PRL secretion in cows [*Bos taurus* (94)], while melatonin oral administration reduces PRL release in both prepubertal (95) and mature (96) cows. The pathway involved in the melatonin-mediated PRL inhibition seems to be mediated through a dopamine-independent mechanism, since melatonin administration inhibits PRL release even in rams where the hypothalamo-pituitary connection has been surgically disrupted (97, 98) and melatonin implants reduce PRL secretion without altering dopamine content in ewe (59).

Despite the involvement of both somatotropes and thyrotropes in seasonal physiological activity, there is no clearly established role for melatonin signaling to their POA/hypothalamic regulators. Somatotrope cells produce GH, a peptide hormone involved in numerous physiological processes including growth, metabolism and cellular proliferation. The main hypothalamic regulators of somatotropes are growth hormone releasing hormone (GHRH) and somatostatin, which stimulates and inhibits GH production, respectively (99). Thyrotrope cells produce TSH, a heterodimeric glycoprotein hormone, composed of an α- (GPHα) and a β- (TSHβ) subunit, involved in different seasonal physiological functions including reproduction and growth (100). Two distinct populations of thyrotropes, with distinct morphology and secretory activity are located in the PT and PD (101–103). Thyrotropin-releasing hormone (TRH) produced by hypothalamic neurons is the main regulator of PD TSH synthesis (104). By contrast, TRH has no effect on PT thyrotrope activity (105), which is controlled by other signals including melatonin, as discussed in section 3 below on direct effects of melatonin.

### Teleosts

**Figure 4:**
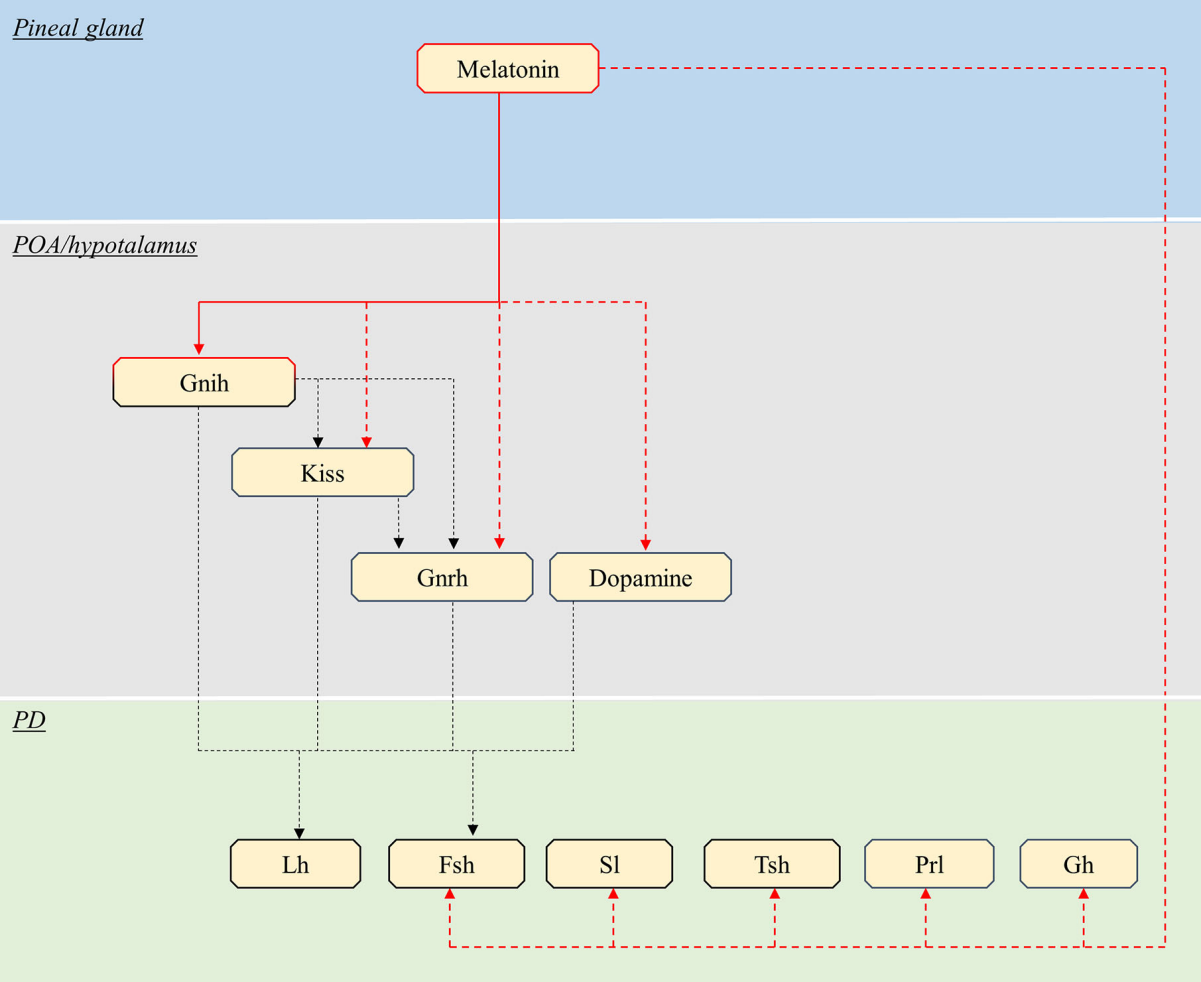
Schematic view of the putative pathways through which melatonin influence pituitary endocrine activity in teleosts. Red continuous lines indicate cell types directly targeted from melatonin. Dashed red lines indicate cells influenced by melatonin *via* yet unidentified interneurons, paracrine signals or Mtnr Note that melatonin might act only on a few of the illustrated pathway, in different species (see text). Black dashed lines indicate all other interactions between brain and pituitary. POA, *preoptic area*; PD, *pars distalis*; Kiss, kisspeptin; Gnrh, gonadotropin-releasing hormone; Lh, luteinising hormone; Fsh, follicle-stimulating hormone; Sl, somatolactin; Tsh, thyroid-stimulating hormone; PRL, prolactin; GH, growth hormone.

In teleosts, endocrine pituitary cells are also mainly controlled by brain signaling centers in the preoptic and hypothalamic areas (41), which are characterized by the presence of numerous melatonin binding sites (106–108). The effects of melatonin on these brain centers, and thus indirectly on pituitary activity, have been studied mainly in the context of reproduction in teleosts, such as in the salmon (109) and eel (110) where melatonin has been shown to play an important role in puberty. Therefore, the available knowledge and thus the discussion in the present review, only concern gonadotropes (**Figure 4**, **Table 2**).

**Table 2:** Summary of the known effects of melatonin POA/hypothalamic neurons controlling pituitary hormonal production in teleosts.

Target	Effect of melatonin	Species	Spawning season/Photoperiod	Description	Reference
**Teleosts**					
Gnrh	Stimulates	Zebrafish	Spring/LP (Daily in captivity)	Melatonin exposure *via* water upregulates brain *gnrh3* expression (adult females)	Carnevali et al. (111)
	Inhibits	Nile tilapia	Spring/LP	Melatonin injections downregulate brain *gnrh1* expression	Kim et al. (112)
		Masu salmon	Autumn/SP	Oral melatonin administration (50 µg/g feed) decreases Gnrh release	Amano et al. (113)
		Sea bass	Spring/LP	Melatonin injections downregulate brain *gnrh1* and *gnrh3* expression	Servili et al. (114)
				Melatonin implants downregulate brain gnrh1 and gnrh3 and gnrhr-II-1a -2b	Alvarado et al. (115)
	None	European eel	Spring/LP	Melatonin implants have no effects on *gnrh* expression	Sébert et al. (116)
Gnih	Stimulates	Nile tilapia	Spring/LP	Melatonin injections upregulate brain *gnih* expression	Kim et al. (112)
	Inhibits	Zebrafish	Spring/LP (Daily in captivity)	Melatonin downregulates *gnih* expression in cultured hypothalamus	Yumnamcha et al. (117)
Kiss	Stimulates	Zebrafish	Spring/LP (Daily in captivity)	Melatonin exposure *via* water upregulates brain *kiss1* and *kiss2* expression (adult females)	Carnevali et al. (111)
	Inhibits	Sea bass	Spring/LP	Melatonin implants downregulate brain *kiss1* and *kiss2* expression	Alvarado et al. (115)
Dopamine	Inhibits	European eel	Spring/LP	Melatonin implants stimulate brain tyrosine hydroxylase activity	Sébert et al. (116)
		Carp	Spring/LP	Melatonin inhibits dopamine release in cultured hypothalamus	Popek (118)
				Melatonin injections inhibit brain dopamine release	Popek et al. (119)
		Asian catfish	Spring/LP	Melatonin inhibits hypothalamic tyrosine hydroxylase activity during preovulatory phase in female	Chaube and Joy (120)
				A higher dose of melatonin has no effect on tyrosine hydroxylase activity	Senthilkumaran and Joy (121)
		Rainbow trout	Autumn/SP	Melatonin decreases hypothalamic-pituitary dopamine turnover	Hernández-Rauda et al. (122)

#### Gonadotropes

In contrast to mammals, the teleost gonadotropes mainly produce Lh and Fsh in distinct cells, with only a small portion of gonadotropes producing both hormones in some species (43). However, like in mammals, pituitary gonadotropin synthesis and release are regulated by POA/hypothalamic signaling centers, including Gnrh, Gnih, Kiss, and dopamine neurons (41, 123, 124).

##### Gnrh

Fish possess up to three genes encoding Gnrh (*gnrh1*, *gnrh2*, *gnrh3*) (16, 41). In some teleost species, melatonin stimulates gonadotropin production by upregulating Gnrh expression. For instance, in adult female zebrafish (*Danio rerio*), melatonin exposure *via* immersion increases the mRNA levels of brain *gnrh3* and pituitary *lhb* (111). In a second study on adult zebrafish females, brain *gnrh3* mRNA levels were increased in both constant light (LL) and constant darkness (DD) as compared to normal light-dark cycles (117). The inconsistent responses of *gnrh* highlight the importance of the experimental conditions, and suggest the presence of different regulatory mechanisms activated by melatonin exposure and photoperiod manipulation, as mentioned in the introduction.

In other teleost species, melatonin inhibits gonadotropin production by downregulating Gnrh expression. In Nile tilapia (*Oreochromis niloticus*), whose development and reproduction are suppressed under SP (125, 126), intraperitoneal melatonin injections simultaneously reduce *gnrh1* mRNA in the brain and both *lhb* and *fshb* mRNA in the pituitary (112). Several studies were performed in male sea bass (*Dicentrarchus labrax*). Both intraperitoneal melatonin injections (114) and melatonin implants (115) downregulate brain mRNA levels of the two hypophysiotropic forms of Gnrh, *gnrh1* and *gnrh3*. Remarkably, these genes show natural daily variations in mRNA levels, with lower levels during the mid-dark phase, when plasma melatonin is highest (114). Melatonin implants also decrease pituitary mRNA levels of Gnrh receptors (named *gnrhr2ba1* and *gnrhr1cb*, according to recent phylogeny, (68) but named *gnrhr-II-1a* and *gnrhr-II-2b*, respectively, in the study), as well as *fshb* (115). Interestingly, pituitary Gnrh1 protein content shows daily variation with minimum levels during night time, under both natural photoperiod and LP (127). While downregulating the Gnrh system, melatonin implants also reduce plasma gonadotropins (Lh and Fsh) and androgens (testosterone, T and 11-keto-testosterone, 11KT) levels, thus impairing sexual maturation (115). Servili and collaborators (128) show that in sea bass, the non-hypophysiotropic Gnrh2 neurons send their projections to the pineal gland, and directly stimulate melatonin secretion. Taken together, these results suggest that melatonin in male sea bass, downregulates the production of the hypophysiotropic Gnrh forms (Gnrh1 and Gnrh3) and their release in the pituitary. This, combined with the reduction of Gnrh receptors in the pituitary, result in gonadotropin downregulation. The use of different concentrations of exogenous melatonin can modulate its effects on Gnrh and gonadotropin content. For example, in underyearling masu salmon (*Oncorhynchus masou*), oral administration of melatonin (50 µg/g feed) under LP increases Fsh and T plasma content but has no effect on Lh (129), suggesting that mimicking SP by melatonin administration stimulates testicular development. However, a 10-fold higher dose (500 µg/g feed) decreases pituitary Gnrh and Lh content together with plasma T levels (113). In contrast, the Gnrh system does not respond to melatonin in some teleost species such as European eel (*Anguilla anguilla*) where melatonin implants had no effects on brain *gnrh1* and *gnrh2* mRNA levels (116).

The specific pathways through which melatonin affects Gnrh are largely unknown. In sea bass, the effects of melatonin on Gnrh neuron activity are most likely mediated *via* interneurons (114), since the distribution of melatonin receptors does not match the distribution of *gnrh1* and *gnrh3* cells (106). In masu salmon, melatonin binding sites were localized in the POA (113), however no colocalization study was performed to investigate their presence in Gnrh neurons.

##### Gnih

Melatonin modulates the activity of Gnih neurons by stimulating or inhibiting Gnih expression in different species. In adult zebrafish females, exogenous melatonin treatment reduces *gnih* mRNA levels in cultured whole brain, while DD decreases *in vivo* brain *gnih* and increases both *lhb* and *fshb* mRNA in the pituitary (117). In contrast, in Nile tilapia (mixed sex), brain *gnih* mRNA levels increase during the night, in parallel with plasma melatonin levels in mature fish (130). Additionally, intraperitoneal melatonin injections increase *gnih* and *mtnr1c* mRNA in the brain and simultaneously decrease *lhb* and *fshb* mRNA in the pituitary.

Kim and colleagues (130) suggest that, like in birds, melatonin might act on Gnih neuron activity *via* Mtnr1c (131). Indeed, in the cinnamon clownfish (*Amphiprion melanopus*), Gnih neurons express Mtnr1c (named from the authors MT-R1) (132). However, whether this is a species-specific case, or a general condition for all teleosts, remains to be investigated.

##### Kiss

In teleosts, contrary to in mammals, Kiss neurons directly regulate pituitary endocrine cells rather than acting through Gnrh neurons (133). Teleosts possess two genes encoding kisspeptins (*kiss1*, *kiss2*) (16, 134). Melatonin is also involved in the control of *kiss* expression in teleosts. In adult female zebrafish, melatonin exposure *via* immersion increases mRNA transcript levels of both *kiss1* and *kiss2* in the brain, and *lhb* in the pituitary (111). While *kiss1* does not respond to photoperiod manipulations, *kiss2* mRNA is induced under LL when melatonin plasma levels are at their minimum (117). In contrast to in zebrafish, prolonged exposure to melatonin *via* implants decreases brain mRNA levels of *kiss1* and *kiss2* in male sea bass (115). The heterogeneity of *kiss* response, as seen for *gnrh*, highlight the influence of experimental conditions and suggest the possible involvement of different pathways influenced by the hormonal and nervous photoperiodic signal.

It is not known whether melatonin acts directly on kiss neurons or operates *via* interneurons in teleosts. In sea bass, Kiss1 and Kiss2 immunoreactive neurons were identified in the lateral tuberal nucleus and parvocellular nucleus, respectively (135), two locations that also express melatonin receptors (106). However, a clear colocalization of the melatonin receptors in Kiss neurons has not been demonstrated.

##### Dopamine

In several teleost species, dopaminergic neurons from the POA exert a strong negative control on gonadotropes, especially Lh-producing cells (82). Melatonin, in turn, influences the activity of hypophysiotropic dopaminergic neurons. In European eel melatonin implants stimulate the dopaminergic system in the POA, increasing tyrosine hydroxylase activity, the rate-limiting enzyme of dopamine synthesis (116). As a consequence, this treatment downregulates both *lhb* and *fshb* mRNA levels. In contrast to the eel, melatonin inhibits the dopaminergic system in other fish species. For instance, in mature female carp (*Cyprinus carpio*), melatonin decreases dopamine release, both in *in vitro* cultured hypothalamus (118) and *in vivo* by direct injection into the third cerebral ventricle (119). The *in vivo* inhibition of dopamine release was registered during the spawning period in summer, but not during sexual regression in winter, suggesting that the effect of melatonin on the dopaminergic system might depend of the maturation stage. Inhibiting effects of melatonin on the dopaminergic systems were also observed in other species, including the threespot wrasse (*Halichoeres trimaculatus*), where intraperitoneal melatonin injections downregulate brain dopamine content (136), and rainbow trout (*Oncorhynchus mykiss*), where melatonin decreases the hypothalamic-pituitary dopamine turnover (122). In preovulatory female catfish (*Heteropneustes fossilis*), melatonin injections for three days inhibit tyrosine hydroxylase enzymatic activity in the hypothalamus (120). However, in a previous study using the same species, reproductive phase and melatonin injection dose, but administrated over a longer period (20 days), failed to affect hypothalamic dopamine turnover (121). This indicates that the length of the treatment with melatonin might affect the response of the dopaminergic system.

Melatonin binding and *mtnr* mRNA distribution studies in rainbow trout (108) indicate that it is very unlikely that Mtnr is present on hypophysiotropic dopaminergic neurons. Therefore, the effects of melatonin might be mediated by interneurons in this species. Studies in Atlantic salmon (137) goldfish (*Carassius auratus*) (138) and Japanese catfish (*Silurus asotus*) (139), identify melatonin binding sites in the POA, where hypophysiotropic dopaminergic neurones are located (82); however, a clear colocalization has not been demonstrated.

### Summary

Melatonin affects pituitary hormonal production in both mammals and teleosts by regulating upstream brain factors. As gonadotropes play a crucial role in the control of reproduction, which often depends on environmental conditions, it is not surprising that especially their response to indirect melatonin signaling has been studied extensively.

In mammals, melatonin modulates gonadotropin expression by acting on POA/hypothalamic signaling centers. It downregulates KISS and GnRH production and stimulate dopaminergic activity in summer breeders, while it upregulates KISS and GnRH production and inhibits dopaminergic activity in winter breeders. Interestingly, melatonin reduces GnIH neuronal activity in both summer and winter breeders, indicating downstream differences in the signaling cascade.

In teleost fish, melatonin affects these brain signaling hubs in a more complex manner, both inhibiting and activating the gonadotrope axis, depending on the species. In some species, melatonin activates the gonadotrope axis by simultaneously stimulating the production of Gnrh and Kiss, while inhibiting Gnih; in others, melatonin exerts a negative action on reproduction. Melatonin may downregulate dopamine production resulting in increased gonadotropin synthesis, or it can stimulate the dopaminergic system and inhibit gonadotropin production. Nevertheless, in both teleosts and mammals, a clear picture of the cell types directly targeted by melatonin in the brain is still scarce. In light of such opposing forces, it is urgent to identify the specific cell types targeted by melatonin in both mammals and teleosts, by determining which ones express MTNR. This is a requirement before being able to fully elucidate the mechanisms involved in the integration of environmental signals in the brain neuroendocrine centers.

## Direct Effects of Melatonin on the Anterior Pituitary

### Mammals

In addition to the effects mediated by the brain, melatonin can act directly on the pituitary gland in mammals (**Figure 3**, **Table 3**).

**Table 3:** Summary of the known effects of melatonin on pituitary *in vitro* and *ex vivo* cultures in mammals.

Species	Type of preparation	Effects of melatonin	Reference
**Mammals**			
Sheep	PT cell culture	Acute: inhibits forskolin-induced secretion of tuberalin	Morgan et al. (140)
	PT cell culture	Acute: inhibits forskolin-induced cAMP	Hazlerigg et al. (141)
	PT cell culture	Prolonged: increase basal and forskolin-induced cAMP	Hazlerigg et al. (141)
	PT cell culture	Reduces *Mtnr1a* mRNA	Barret et al. (142)
	PT explants	Reduces *Mtnr1a* mRNA	Fustin et al. (143)
	PT explants and cell culture	Reduces *Egr1* expression	Fustin et al. (143)
Rat (neonatal)	PD organ cultures	Inhibits LH and FSH release	Martin and Klein (144) Martin and Sattler (145)
	PD cell culture	Inhibits GnRH-induced Ca2+ signal and LH secretion	Vaněček and Klein (146)
	PD cell culture	Inhibits GnRH-induced cFOS	Sumova et al. (147)
	Organ cultures	No effect on TRH-induced TSH/PRL or SRIF-induced inhibition of GH	Martin and Klein (144) Martin and Sattler (145)
Rat (maturing)	PD cell culture	No effect on GnRH-response	Rivest et al. (148)
Rat (Adult)	TP/ME explants	Inhibits Lh release	Nakazawa et al. (149)
	GH3/GH4 cell line	Inhibits secretion of PRL and GH (no effect on cAMP)	Griffiths et al. (150)
	GH3 cell line	Inhibits basal and forskolin-induced PRL secretion and expression	Ogura-Ochi et al. (151)
Baboon	PD cell culture	No effect on LH or FSH secretion	Ibáñez-Costa et al. (152)
Baboon	PD cell culture	Stimulates GH and PRL expression and release	Ibáñez-Costa et al. (152)
	PD cell culture	Increases expression of receptors for GhRH and ghrelin, decreases receptors for somatostatin	Ibáñez-Costa et al. (152)
	PD cell culture	No effect on ACTH or TSH	Ibáñez-Costa et al. (152)
Mouse	ATt20 cell line	Inhibits cAMP and ACTH release	Tsukamoto et al. (153)

#### Melatonin Receptors in Mammalian Pituitary

The main target for melatonin within the mammalian pituitary is the PT, as indicated by the important presence of melatonin binding sites in all mammalian species investigated so far. Those include, for instance, Siberian hamster (154, 155); Syrian hamster, (154–156); rat, (154, 155, 157, 158); red deer (*Cervus elaphus*) (159); ferret (*Mustela putorius furo*) (160), rhesus monkeys (*Macaca mulatta*) (161) and human (89). MTNR1A (MT1) is the main form of melatonin receptor present in the PT. *Mtnr1a* mRNA was detected by *in situ* hybridization in the PT of sheep, Siberian hamster and rat (154) and in primary PT cell cultures of sheep (162). MTNR1A has also been detected in human PT, *via* immuno-staining (89). Identification of the specific cell types expressing melatonin receptors is a key requisite to discriminate the direct effects mediated by melatonin. Double labelling combining *in situ* hybridization and immunohistochemistry shows that Mtnr1a is expressed in most, but not all, thyrotrope cells within the PT, while it is absent from the other endocrine cells types in the anterior pituitary in European hamster (*Cricetus cricetus*) (163) and rat (157). This suggests that thyrotropes of the PT are the main pituitary target of melatonin action in mammalian species.

Interestingly, the expression of Mtnr within the PT varies across the year, in response to different neuroendocrine factors (34), modulating the endocrine response to melatonin between the different seasons. For instance, Mtnr expression increases during the reproductive season, peaking under LP in summer breeders or SP in winter breeders. Simulating winter season in the summer breeder Syrian hamster, using artificial SP (164) or melatonin injections (156, 165) induces a marked decrease in MTNR density in the PT of Syrian hamster. Melatonin injections decrease MTNR1A density in both Syrian and Siberian hamster PT (156). Similar effects of SP were shown in European hamster (163, 166) and hedgehog*, Erinaceus europaeus* (167). On the contrary, in winter breeders such as the mink (*Mustela vison*), the density of melatonin binding sites in the PT plummets in July and peaked in October, in concomitance with reactivation of sexual activity (168).

Melatonin and the pituitary also regulate daily physiological rhythms. As a consequence, variations in the presence of MTNR within the PT are not limited to annual cycles, but also occur within the span of a single day. Mtnr1a mRNA levels vary during the day in the PT of Siberian and Syrian hamster, peaking during daytime and decreasing at night (156).

Finally, in the PD of foetal rat, *Mtnr1a* mRNA was also identified by *in situ* hybridization before activation of the GnRH system, but not in postnatal stages (169). Bae and colleagues (170) demonstrate that *Mtnr1a* transcripts are present in the mice αT3-1 gonadotrope cell line, but their expression is downregulated after exposure to GnRH. The activation of the GnRH system in postnatal stages might therefore be responsible for the lack of MTNR in the adult PD reported in rat. Melatonin binding sites were also detected in the PD of adult sheep (171, 172), red deer (159) and ferret (160). A weak staining of *MTNR1A* was detected in human PD by *in situ* hybridization (89). To date, the identity of the cellular targets of melatonin within the PD and their contribution to the hormonal regulation of the gland remain largely unknown.

#### Effects of Melatonin on the *Pars Tuberalis*

**Figure 5:**
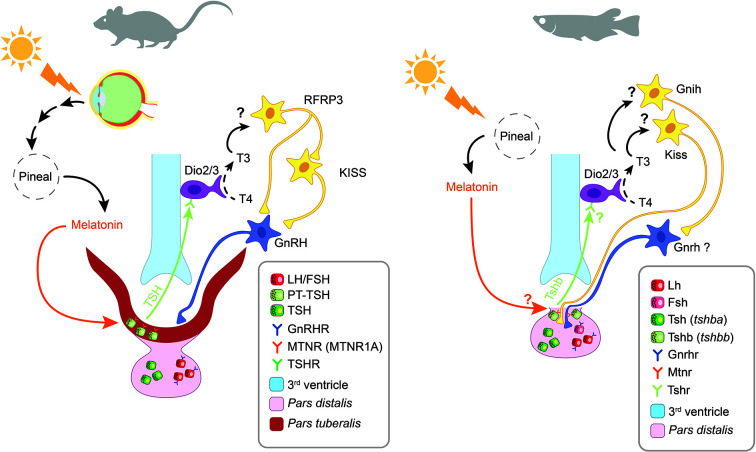
Melatonin-induced retrograde signaling in mammals and teleosts. Thyrotrope and gonadotrope cells are respectively represented as green and red squares (see legend). Question marks (?) indicate putative pathways not yet demonstrated. In mammals, the photic signal perceived from the retina reaches the pineal gland after being processed from different brain centers (including the suprachiasmatic nucleus, SCN) thus regulating the rhythmic release of melatonin at night. Circulating melatonin acts on *pars tuberalis* (PT) thyrotropes (PT-TSH) *via* MTRN1A, thus inhibiting PT-TSH release. In spring, when melatonin levels decrease, PT-TSH secretion is stimulated. PT-TSH, guided by tissue specific glycosylation, binds on its receptors on tanycytes located in the third ventricle of the hypothalamus. Here, PT-TSH regulates local deiodinases (Dio2/Dio3) influencing thyroid hormone metabolism by promoting the conversion of T4 into the bioactive T3. T3 in turn activates *arcuate nucleus* (ARC) kisspeptin (KISS) neurons *via* a still unknown mechanism. The following increase in gonadotropin releasing hormone (GnRH) release, stimulates gonadotropes activity in the *pars distalis* (PD). In teleosts the photic signal is directly perceived from photoreceptive structures within the pineal gland, thus regulating the rhythmic release of melatonin at night. Recent studies suggest that melatonin might regulate the release of a retrograde signal from the pituitary also in teleosts. A distinct population of thyrotrope cells (expressing a second Tsh paralogue, *tshbb)* located near the pituitary stalk, drastically increase *tshbb* expression under long photoperiod, a similar response to the one occurring in mammalian PT-TSH. Although melatonin receptors have been described in teleost pituitary and found to display daily and seasonal regulation, their presence in this thyrotrope population as well as the inhibition of Tsh synthesis and release in response to melatonin, remain to be demonstrated.

As mentioned above, melatonin pituitary binding primarily takes place in the PT. Through these cells, melatonin seems to regulate the PD activity *via* at least two different routes, a retrograde route from the PT to the brain (**Figure 5**) and an anterograde route from the PT to the PD, as discussed below.

##### Retrograde Route

Studies in rodents demonstrate the inhibitory effects of melatonin on PT-TSH synthesis (173) *via* MTNR1A (174). During the long days in spring/summer, PT-TSH rises independently from TRH stimulation (175) due to reduced circulating melatonin levels. In the retrograde route (**Figure 5**), the expression of PT-TSH is rapidly induced, after LP exposure, by the transcription factor EYA3, which works with the circadian transcription factor thyrotroph embryonic factor (TEF) (176, 177). Melatonin acutely inhibits *Eya3* expression, but at the same time induces a peak of *Eya3* 12 h later. This leads to a strong morning peak of *Eya3* (and subsequently TSH) during long days. Although several other collaborating transcription factors are involved in the precise circannual regulation of TSH secretion from PT, EYA3 seems to be the one regulated by photoperiod *via* melatonin.

PT-TSH then reaches the brain where it binds to its receptors in tanycytes, specialized ependymal cells within the mediobasal hypothalamus, thus regulating the enzymatic activity of local deiodinases (Dio2-Dio3) (173, 178). This controls local thyroid hormone (TH) metabolism by converting thyroxine (T4) to the bioactive triiodothyroxine (T3), serving as key regulator of seasonality (173). Ikegami and collaborators (175) demonstrate that specific post-translational glycosylations allow PT-TSH to exclusively target the hypothalamus, and not the thyroid. While the cellular and molecular targets remain to be clearly identified, recent findings indicate that the increase of T3 in the mediobasal hypothalamus acts on KISS1 and RFRP3 neurons, which in turn modulate GnRH secretion (78). The molecular pathway from melatonin to T3 production appears to be conserved in mammalian species regardless of their reproductive strategy as summer or winter breeders (67, 179). Therefore, species-specific differences might occur downstream of this common pathway.

##### Anterograde Route

In the anterograde route, melatonin regulates PRL synthesis and secretion in the PD by inhibiting the release from the PT of one or more PRL-releasing factors named “tuberalin” (67). To date, the PT-specific factor(s) are still undetermined, as more than 30 different factors are known to stimulate PRL secretion (92). Several candidates have been proposed including tachykinin-1 and neurokinin A in sheep (180) or endocannabinoids in hamster (179, 181). Notably, these factors might act through folliculo-stellate cells to regulate lactotropes (182).

Tuberalin secretion can indeed be stimulated in ovine PT cell cultures by forskolin (140), an activator of adenylyl cyclase (AC), the enzyme catalysing the conversion of ATP to cyclic AMP (cAMP). The forskolin-induced secretion of tuberalin from PT cells was assessed by adding medium from the PT culture to a PD culture and measuring the amount of PRL secreted in response. Melatonin acutely inhibited the forskolin-induced secretion of tuberalin but had no effect alone. In support, melatonin inhibited the forskolin-induced cAMP production in ovine PT cells (141). Furthermore, melatonin downregulated the expression of its own receptor in PT cells from rat (183) and sheep (142). In ovine PT cells, the downregulation of *Mtnr* expression involves the cAMP signaling pathway (143). Together, these results imply that melatonin works through the MTNR/Gi/cAMP pathway to inhibit tuberalin secretion and subsequently regulating PRL production in the PD. Interestingly, incubation with melatonin for 16 h sensitizes AC, increasing both basal and forskolin-induced cAMP production (141). After the prolonged melatonin exposure, acute application of melatonin still inhibits the forskolin-induced cAMP increase.

For melatonin to inhibit secretion of tuberalin, there must be a stimulating factor that melatonin can oppose. This “tuberalin releasing factor” has not been identified but was named StimX by Morgan and Williams (184). Dardente and colleagues (67) proposed dopamine as a promising candidate for StimX, arguing that it might act through the dopamine receptor D1 expressed in PT cells, whose activation increases the intracellular cAMP level in neurons.

Downstream of cAMP, melatonin up-regulates or down-regulates the expression of a range of genes (67). Several of them are clock genes, including Period1 (*Per1*) and Cryptochrome-1 (*Cry1*) (185). This implies that PT cell activity might be regulated by an internal clock and that the clock itself may be modulated by melatonin. Interestingly, the same clock genes are not affected by melatonin in the suprachiasmatic nucleus (SCN) of the hypothalamus, indicating that the SCN clock is working more independently than peripheral clocks (186–188). In ovine PT cell cultures and explants, the expression of the immediate early gene *Egr1* is acutely suppressed by melatonin, which otherwise follows a daily rhythm (143). EGR1 in turn regulates several genes, some being upregulated, such as *Cry1*, others downregulated such as *Mrnt1a*. In contrast to *Mtnr1a*, the expression of *Cry1* was not affected by changes in cAMP levels.

Other PT endocrine cells beside thyrotropes might also be regulated by melatonin. Nakazawa and collaborators (149) found that melatonin inhibits LH release from male rat tissue explants (consisting of PT and median eminence) in a dose-dependent manner. This in turn increases the release of GnRH from the median eminence part of the explant.

#### Effects of Melatonin on the *pars distalis*

##### Gonadotropes

In rodents, the effects on PD gonadotropes seem highly age-specific, with clear inhibitory effects in neonatal animals and no effects in adults. Melatonin (1–10 nM) reduces the GnRH-induced LH and FSH release in pituitary organ cultures from neonatal rats (144, 145). Furthermore, Vanecek and Klein (146) demonstrate that melatonin (10 nM) reduces the GnRH-induced Ca^2+^ signal and subsequently LH secretion. Similarly, Pelisek and Vanecek (189) demonstrated that melatonin (2 nM) reduces GnRH-induced LH release, as well as the forskolin-induced cAMP production, in cell cultures from neonatal rats. Melatonin (1 nM) directly inhibits GnRH-induced Ca^2+^ signaling in neonatal gonadotropes, both *via* plasma membrane Ca^2+^ channels and endoplasmic reticulum Ca^2+^ release channels (190). The inhibitory effect of melatonin on the GnRH-induced Ca^2+^ oscillations might not be uniform over the gonadotrope cell population as the responses differ between cells, indicating a complex regulatory pathway (191, 192). The role of MTNR in neonatal PD may not be limited to gonadotrope regulation. In light of the previously described role of melatonin on the regulation of clock genes, Johnston and colleagues (169) suggest that *Mtnr1a* expression may reflect a developmental requirement for circadian synchronization between tissues before mature regulatory pathways become established. Additionally, the promoter region of rat *Mtnr1a* contains response elements for transcription factors involved in pituitary cell differentiation and regulation (169). Melatonin might therefore be involved in the correct development of the embryonic PD. Indeed, melatonin (100 nM) inhibits GnRH-induced increase of *cFos* (a proto-oncogene involved in cellular proliferation and differentiation) immunoreactivity in neonatal rat pituitary PD culture (147).

During development and maturation, melatonin binding is reduced, and in adults, melatonin does not have the same direct effect on the pituitary. Rivest and collaborators (148) found that melatonin incubation (5 nM) of pituitary cell cultures from sexually maturing rats does not modify the GnRH response. Likewise, Ibáñez-Costa and colleagues (152) found no effect of melatonin (pM to µM range) on the FSH and LH secretion in primary pituitary cultures from adult female baboons.

##### Other Endocrine Cells in the pars distalis

Regarding the effects of melatonin on other PD endocrine cells, the results are even scarcer. Melatonin (10^-8^ to 10^-6^ M) reduces the production and secretion of both PRL and GH from the rat pituitary cell line GH4C1, but has no effect on basal or stimulated cAMP levels (150). Similarly, Ogura-Ochi and collaborators (151) show that melatonin suppresses both basal and forskolin-induced PRL secretion and mRNA abundance in the closely related GH3 cell line. In contrast, in primary pituitary cell cultures from adult female baboons, melatonin increases GH and PRL expression and release in a dose-dependent manner, an effect blocked by somatostatin (152). Both the common (AC/PKA/Ca-channels) and distinct (PLC/Ca-release) pathways seem to be involved. Melatonin (10 nM) also affects the expression of GHRH receptors, ghrelin and somatostatin, but not expression or release of ACTH or TSH. Also in pituitary organ cultures from neonatal rats, melatonin has no effect on TRH-induced TSH/PRL release or somatostatin-induced inhibition of GH release (144, 145). In the mice corticotrope cell line AtT20, melatonin reduces the levels of ACTH, alongside a reduction in cAMP (153).

### Teleosts

As in mammals, melatonin can also act directly on the pituitary gland in teleosts (**Figure 4**, **Table 4**).

**Table 4:** Summary of the known effects of melatonin on pituitary *in vitro* and *ex vivo* cultures in teleosts.

Species	Type of preparation	Effects of melatonin	Reference
**Teleosts**			
Goldfish	Primary cell culture	No effect on AC activity	Deery et al. (193)
	Perfused fragments	No effect on secretion of Lh, Fsh or Gh.	Somoza and Peter (194)
European eel	Primary cell culture	No effect on *fshb* and *lhb* mRNA levels	Sébert et al. (116)
Atlantic croaker	Perfused fragments	Stimulates Lh (GthII) secretion	Khan and Thomas (195)
Pike	Whole pituitary	Inhibits forskolin-induced cAMP	Gaildrat and Falcón (196)
Rainbow trout	Primary cell culture	Low dose: inhibits forskolin-induced cAMP and Gh secretion	Falcón et al. (197)
	Primary cell culture	High dose: stimulates Gh secretion	Falcón et al. (197)
	Primary cell culture	High dose: inhibits Prl secretion	Falcón et al. (197)
European sea bass	Primary cell culture	Increases *Cry1* and *Cry2* mRNA levels	Herrero and Lepesant (198)
Medaka	Whole pituitary organ culture	Reduces *fshb*, *tshb* and *sl* mRNA levels (not *lhb*, *gh*, *prl* or *pomc)*	Kawabata-Sakata et al. (199)

#### Melatonin Receptors in Teleosts Pituitary

Multiple Mtnr paralogues are expressed in the pituitary of teleosts (28). For instance, qPCR analysis detected the mRNA of four Mtnr paralogues in the pituitary of medaka (27). Three were described in Senegalese sole (*Solea senegalensis*) (200), goldfish (201), and Atlantic salmon (25). Two were detected in chum salmon (*Oncorhynchus keta*) (202) and pike (*Esox lucius*) (196). One was detected in European sea bass (203), suggesting possible multiple effects of melatonin which might also vary between species.

The exact location of melatonin receptor/binding sites in teleosts is not clear. Despite the aforementioned identification of *mtnr* mRNA in goldfish pituitary, Martinoli and colleagues (107) observe no specific binding of melatonin. On the other hand, rainbow trout (197) and pike (196) pituitaries have 2-[^125^I]iodo-melatonin binding sites. However, the assay used was aimed at characterizing the binding capacity rather than their localization within the pituitary, although a regional distribution was reported, with binding sites clustering together in close proximity.

Like in mammals, the abundance of pituitary Mtnr in teleosts varies with the season and the day, suggesting a correlation with physiological state. The Senegalese sole (200) shows seasonal fluctuations with higher *mtnr* mRNA levels during the summer spawning period. While a first study in Atlantic salmon carried out in autumn indicated the absence of melatonin binding sites (137), a later study shows that Mtnr exhibits both seasonal and daily fluctuations in the male parr pituitary (25). In spring during sexual maturation, pituitary *mtnr* mRNA peaks during the day and drops at night, while in autumn minimal levels are constantly maintained throughout the day. In medaka, where SP inhibits reproduction, pituitary *mtnr1a, mtnr1c*, and *mtnr1d*, but not *mtnr1b* mRNA levels show daily fluctuations with higher levels at night under LP but not SP (27). The presence of multiple Mtnr paralogues whose concentrations vary across the season indicates a complex role of melatonin in teleost pituitary physiology. It is not yet clear precisely which cell types express *mtnr* in teleosts. Indeed, the seasonal and daily variations in *mtnr* mRNA levels observed in sole, sea bass, salmon, and medaka suggest a relation with the reproductive status of the fish. These fluctuations also highlight the importance of both the timing of sampling and the application of different techniques for the successful identification of the cells expressing Mtnr within the pituitary.

#### Effects of Melatonin in Teleosts Pituitary

##### Putative Retrograde Signal

Unlike mammals, teleosts do not possess an anatomically distinct PT. Nakane and Yoshimura (204) propose that, in salmonids, the translation of the photoperiodic signals into pituitary hormonal messages might instead take place in the *saccus vasculosus* (SV), a secretory organ located posterior to the pituitary and directly connected to the third ventricle of the hypothalamus. In masu salmon, specialized cells within the SV, the coronet cells, possess all the components involved in the regulation of seasonal reproduction *via* the Tsh pathway (Tshβ, Tsh receptors and Dio2). However, the exact photoperiodic role of the SV (and the involvement of melatonin in it), remains controversial. First, the SV signal pathway is presumably directly activated by photo-transduction through photoreceptive pigments without the requirement of melatonin (205). Additionally a recent study in juvenile Atlantic salmon reported no photoperiodic effects on SV *tshb* and *dio2b* mRNA levels (206). Finally, the SV is not present in all fish [e.g. it is absent in zebrafish (207, 208)]. Therefore, a different mechanism might be involved in the photoperiodic control of seasonality in fish.

Several teleosts possess two *tshb* paralogues resulting from the 3R, *tshba* and *tshbb* (209) (named by the authors *tshβ* and *tshβ3*). In Atlantic salmon, both are expressed in the pituitary, but only *tshbb* is expressed in the SV (210). Interestingly, while pituitary *tshba* mRNA levels are relatively constant, *tshbb* mRNA level vary profoundly, with a peak concomitant with the onset of downstream migration, in spring. Since the two paralogs also show expression at distinct locations in the pituitary, Fleming and colleagues (210) propose that *tshbb-*expressing cells, located near the pituitary stalk, are analogous to the mammalian PT-TSH cells and possibly regulate the retrograde diffusion of Tsh to the hypothalamus (**Figure 5**).

Irachi and collaborators (206) demonstrate a significant increase in pituitary *tshbb* mRNA levels followed by a rise in *dio2b* mRNA levels in the midbrain/optic tectum and hypothalamus in response to increased daylength, providing additional evidence that the pituitary Tshb (formed by the *tshbb* subunit) is a key contributor to photoperiodic signaling in fish, similar to the mammalian PT-TSH. However, whether like in mammals, melatonin signal is directly integrated by the pituitary Tshb (*tshbb-*expressing) cells in teleosts, is not known.

##### Other Endocrine Cells in the Pars Distalis

The direct effects of melatonin on other endocrine cell types have not been extensively investigated in teleosts and the few studies available indicate different effects between species.

In goldfish, melatonin (10^-6^–10^-3^ M) has no effect on AC activity in homogenized pituitary samples (193). Although AC is part of the most common signaling pathway triggered by melatonin receptors, this result does not rule out the possibility that melatonin may trigger effects *via* other pathways. The conclusion that melatonin most probably had no direct effect on the pituitary was further supported by a study on perfused goldfish pituitary fragments, from which melatonin did not affect secretion of gonadotropins or Gh (194). Similarly, Sébert and colleagues (116) report from unpublished *in vitro* experiments that melatonin has no effect on *fshb* and *lhb* mRNA levels in primary pituitary cultures from European eel.

However, effects of melatonin have been observed in other species. Melatonin (0.2 ng/ml) stimulates Lh (GtH II) secretion from pituitary fragments from mature Atlantic croaker (195). In trout pituitary organ- and cell cultures, high concentration of melatonin [close to the night-time circulating levels as determined by Gern and colleagues (211)] induces a dose-dependent Gh secretion in the absence of forskolin, along with a decrease in the secretion of Prl (197). In contrast, low melatonin concentration (close to daytime circulating levels) inhibits the forskolin-induced increase in cAMP levels and Gh secretion. Finally, melatonin exposure (10^-5^ M) in *ex vivo* whole pituitary organ culture from sexually mature medaka, decreases *fshb*, *tshba* and *sl* mRNA levels but has no significant effects on *lhb*, *gh*, *prl*, and *pomc*, (199).

### Summary

The presence of melatonin receptors indicates that melatonin can directly regulate pituitary cells. According to the receptor localization, the PT thyrotropes are the main targets of melatonin in the mammalian pituitary. A few mammalian species also have melatonin binding sites in the PD, suggesting a possible direct control of melatonin on PD endocrine production. The teleost pituitary also expresses several Mtnr paralogues, however their localization is not clear.

In mammals, most of the direct effects of melatonin on the pituitary take place in the PT, in particular on thyrotropes (**Figure 5**). Here, melatonin acting *via* MTNR1A (MT1) affects the expression levels of a set of clock genes that in turn regulate synthesis and secretion of TSH and the hitherto unidentified tuberalin, for retrograde and anterograde seasonal regulation of gonadotropes and lactotropes in the PD, respectively. The signaling pathway from MTNR1A activation to transcriptional regulation seems to mainly be *via* inhibition of AC/cAMP and possibly CREB. Studies on the direct effects of melatonin in the PD are still scarce, but there is solid evidence that melatonin inhibits LH secretion from PD gonadotropes in neonatal stage, but not in adults.

Fewer studies on the direct effects of melatonin on the teleost pituitary have been performed. They report different effects depending on the species and the experimental conditions. Nevertheless, recent works described a new pituitary Tsh population responding to variation in photoperiod similarly to PT-TSH in mammals, suggesting that a Tsh retrograde signaling might also occur in teleosts. It is therefore crucial that future research investigate the presence of melatonin receptors and the responsiveness to melatonin in this cell type to verify whether, similar to in mammals, melatonin signal is directly integrated by Tshb (*tshbb-*expressing) cells in teleosts.

## Discussion

As shown in the present review, the response of the pituitary to melatonin is highly plastic and differs between seasons, time of the day, physiological status, and reproductive strategies. The comparison between mammals and teleosts reveals a greater knowledge gap in the latter group, leaving many open questions on the role of melatonin in regulating the pituitary hormonal production. In both mammals and teleosts however, the exact pathways and cell types targeted by melatonin are largely unknown. It is therefore crucial to clearly describe the integration of the melatonin signal, and identify the cell types expressing melatonin receptors, in the pituitary as well as in the brain. Multi-color *in situ* hybridization or immuno-labelling and single cell transcriptomic approaches are relevant techniques that can be applied to identify the cell types directly targeted by melatonin. To validate the effects of melatonin on the different cell types a combination of *in vivo*, *ex vivo* and *in vitro* studies will be necessary, as no single approach can produce a complete and reliable picture by itself. For instance, caution should be taken when investigating dissociated endocrine pituitary cells in culture, as a recent study shows that dissociation leads to a quick cellular phenotypic change in the pituitary cells (212). Additionally, when deciding on animal model and experimental conditions, one should also consider the important biological differences that might influence, or be influenced by, a time-keeping hormone as melatonin, such as nocturnal versus diurnal habits, different reproductive seasons or hibernation periods. Indeed, the response in animals adapted during numerous generations to stable laboratory conditions, such as mice, rats, zebrafish and medaka, might diverge from the ones in the wild.

In addition, given the seasonality of the processes regulated by melatonin, the localization of MTNR appears to be heavily influenced by factors such as physiological status, season, but also time of the day. It is therefore reasonable to assume that the expression of MTNR in some key cell types might be characteristic of specific physiological conditions and might still remain undetected when analyzed outside a particular time window.

Finally, when investigating the effects of melatonin on neuroendocrine system activity, teleosts show a remarkable plasticity as their response is more sensitive to variation in environmental conditions as compared to mammals (1, 213). As a consequence, it is harder to draw a general picture of the role of melatonin on teleosts brain and pituitary, as the choice of the season or the time of the day to perform the experiment appear to influence the response of the pituitary gland in teleosts.

Because of this sheer number of physiological and environmental variables, as well as significant inter-specific variation, unravelling the full impact of melatonin on the pituitary gland remains a challenge.

## Author Contributions

EC, TH, and RF contributed to the conception and design of the review. All authors contributed to the article and approved the submitted version.

## Funding

This work was supported by the Norwegian University of Life Sciences (NMBU), Ås, Norway, and the University of Oslo (UiO), Oslo, Norway, with the support of the Marie Curie Alumni Association.

## Conflict of Interest

The authors declare that the research was conducted in the absence of any commercial or financial relationships that could be construed as a potential conflict of interest.

## Abbreviations

1R to 4R, 1st to 4th whole genome duplication; AANAT, Arylalkylamine N-acetyltransferase; AC, Adenylyl cyclase; ACTH, Adrenocorticotropic hormone; ARC, Arcuate nucleus; ATP, Adenosine 5’-triphosphate; cAMP, Cyclic adenosine 5’-monophosphate; cGMP, Cyclic guanosine monophosphate; CREB, Calcium/cAMP response element binding protein; Cry1, Cryptochrome1; DIO2/3, Deiodinase 2/3; ER, Endoplasmatic reticulum; EYA, Eyes absent homologue; FSH/FSHB, Follicle-stimulating hormone/FSH beta subunit; GH, Growth hormone; GnIH, Gonadotropin inhibiting hormone; GnRH, Gonadotropin releasing hormone; GnRHR, Gonadotropin releasing hormone receptor; LP/SP, Long photoperiod/Short photoperiod; LH/LHB, Luteinizing hormone/LH beta subunit; LL/DD, Constant light/Constant darkness; ME, Median eminence; MSH, Melanocyte-stimulating hormone; MTNR, Melatonin receptors; PD, Pars distalis; Per1, Period1; PI, Pars intermedia; PKA, Protein kinase A; PKC, Protein kinase C; PLC, Phospholipase C; POA, Preoptic area; POMC, Pro-opiomelanocortin; PRL, Prolactin; PT, Pars tuberalis; RFRP, RFamide related peptide; SCN, Suprachiasmatic nucleus; SL, Somatolactin; T3, Triiodothyroxine; T4, Thyroxine; TEF, Thyrotroph embryonic factor; TH, Thyroid hormone; TRH, Thyrotropin-releasing hormone; TSH/TSHB, Thyroid stimulating hormone/TSH beta subunit.

**Gene and protein nomenclature:** The present review follows the ZFIN nomenclature conventions for protein and gene names in mammals and fish (e.g. Mammalian protein: LHB; Mammalian gene: Lhb; fish protein: Lhb; fish gene: lhb).

## References

[1] Falcón J Zohar Y Photoperiodism in fish. In: Encyclopedia of Reproduction. New York (USA): Elsevier (2018). p. 400–8. 10.1016/B978-0-12-809633-8.20584-0

[2] Reiter RJ Tan D-X Sharma R Historical Perspective and Evaluation of the Mechanisms by which Melatonin Mediates Seasonal Reproduction in Mammals. Melatonin Res (2018) 1(1):59–77. 10.32794/mr11250004

[3] Reiter RJ Melatonin: The chemical expression of darkness. Mol Cell Endocrinol (1991) 79(1-3):C153–8. 10.1016/0303-7207(91)90087-9 1936532

[4] Falcón J Cellular circadian clocks in the pineal. Prog Neurobiol (1999) 58(2):121–62. 10.1016/S0301-0082(98)00078-1 10338357

[5] Klein DC Coon SL Roseboom PH Weller JL Bernard M Gastel JA The Melatonin Rhythm-generating Enzyme: Molecular Regulation of Serotonin N-acetyltransferase in the Pineal Gland. Recent Prog Horm Res (1997) 52:307–57. 9238858

[6] Klein DC Arylalkylamine N-acetyltransferase: “The timezyme.” J Biol Chem (2007) 282(7):4233–7. 10.1074/jbc.R600036200 17164235

[7] Klein DC The 2004 aschoff/pittendrigh lecture: Theory of the origin of the pineal gland - A tale of conflict and resolution. J Biol Rhythms (2004) 19(4):264–79. 10.1177/0748730404267340 15245646

[8] Klein DC Evolution of the vertebrate pineal gland: The AANAT hypothesis. Chronobiol Int (2006) 23(1-2):5–20. 10.1080/07420520500545839 16687276

[9] Falcón J Coon SL Besseau L Cazaméa-Catalan D Fuentès M Magnanou E Drastic neofunctionalization associated with evolution of the timezyme AANAT 500 Mya. Proc Natl Acad Sci USA (2014) 111(1):314–9. 10.1073/pnas.1312634110 PMC3890824 24351931

[10] Putnam NH Butts T Ferrier DEK Furlong RF Hellsten U Kawashima T The amphioxus genome and the evolution of the chordate karyotype. Nature (2008) 453(7198):1064–71. 10.1038/nature06967 18563158

[11] Simakov O Marlétaz F Yue JX O’Connell B Jenkins J Brandt A Deeply conserved synteny resolves early events in vertebrate evolution. Nat Ecol Evol (2020) 4(6):820–30. 10.1038/s41559-020-1156-z PMC7269912 32313176

[12] Dehal P Boore JL Two rounds of whole genome duplication in the ancestral vertebrate. PLoS Biol (2005) 3(10):e314. 10.1371/journal.pbio.0030314 16128622 PMC1197285

[13] Amores A Force A Yan YL Joly L Amemiya C Fritz A Zebrafish hox clusters and vertebrate genome evolution. Science (80- ) (1998) 282(5394):1711–4. 10.1126/science.282.5394.1711 9831563

[14] Allendorf FW Thorgaard GH Tetraploidy and the Evolution of Salmonid Fishes. In: Evolutionary Genetics of Fishes. Boston, MA: Springer US (1984). p. 1–53. 10.1007/978-1-4684-4652-4_1

[15] Ohno S Muramoto J Christian L Atkin NB Diploid-tetraploid relationship among old-world members of the fish family Cyprinidae. Chromosoma (1967) 23(1):1–9. 10.1007/BF00293307

[16] Dufour S Quérat B Tostivint H Pasqualini C Vaudry H Rousseau K Origin and Evolution of the Neuroendocrine Control of Reproduction in Vertebrates, With Special Focus on Genome and Gene Duplications. Physiol Rev (2020) 100(2):869–943. 10.1152/physrev.00009.2019 31625459

[17] Li J You X Bian C Yu H Coon SL Shi Q Molecular evolution of aralkylamine n-acetyltransferase in fish: A genomic survey. Int J Mol Sci (2015) 17(1):51. 10.3390/ijms17010051 PMC4730296 26729109

[18] Cazaméa-Catalan D Besseau L Falcón J Magnanou E The timing of timezyme diversification in vertebrates. PLoS One (2014) 9(12):112380. 10.1371/journal.pone.0112380 PMC4259306 25486407

[19] Coon SL Klein DC Evolution of arylalkylamine N-acetyltransferase: emergence and divergence. Mol Cell Endocrinol (2006) 252(1-2):2–10. 10.1016/j.mce.2006.03.039 16697105 PMC1578506

[20] Paulin CH Cazaméa-Catalan D Zilberman-Peled B Herrera-Perez P Sauzet S Magnanou E Subfunctionalization of arylalkylamine N-acetyltransferases in the sea bass *Dicentrarchus labrax*: two-ones for one two. J Pineal Res (2015) 59(3):354–64. 10.1111/jpi.12266 26267754

[21] Borjigin J Li X Snyder SH The pineal gland and melatonin: molecular and pharmacologic regulation. Annu Rev Pharmacol Toxicol (1999) 39:53–65. 10.1146/annurev.pharmtox.39.1.53 10331076

[22] Bayarri MJ Rol de Lama MA Madrid JA Sanchez-Vazquez FJ Both pineal and lateral eyes are needed to sustain daily circulating melatonin rhythms in sea bass. Brain Res (2003) 969(1-2):175–82. 10.1016/s0006-8993(03)02297-2 12676378

[23] Muñoz-Pérez JL López-Patiño MA Álvarez-Otero R Gesto M Soengas JL Míguez JM Characterization of melatonin synthesis in the gastrointestinal tract of rainbow trout (*Oncorhynchus mykiss*): distribution, relation with serotonin, daily rhythms and photoperiod regulation. J Comp Physiol B Biochem Syst Environ Physiol (2016) 186(4):471–84. 10.1007/s00360-016-0966-4 26873742

[24] Brydon L Petit L de Coppet P Barrett P Morgan PJ Strosberg AD Polymorphism and signalling of melatonin receptors. Reprod Nutr Dev (1999) 39(3):315–24. 10.1051/rnd:19990304 10420434

[25] Ciani E Fontaine R Maugars G Mizrahi N Mayer I Levavi-Sivan B Melatonin receptors in Atlantic salmon stimulate cAMP levels in heterologous cell lines and show season-dependent daily variations in pituitary expression levels. J Pineal Res (2019) 67(3):e12590. 10.1111/jpi.12590 31169933

[26] Denker E Ebbesson LOE Hazlerigg DG Macqueen DJ Phylogenetic reclassification of vertebrate melatonin receptors to include Mel1d. G3 (Bethesda) (2019) 9(10):3225–38. 10.1534/g3.119.400170 PMC6778780 31416806

[27] Sakai K Yamamoto Y Ikeuchi T Vertebrates originally possess four functional subtypes of G protein-coupled melatonin receptor. Sci Rep (2019) 9(1):9465. 10.1038/s41598-019-45925-2 31263128 PMC6602942

[28] Maugars G Nourizadeh–lillabadi R Weltzien F-A Nourizadeh-Lillabadi R Weltzien F-A New insights into the evolutionary history of melatonin receptors in vertebrates, with particular focus on teleosts. Front Endocrinol (Lausanne) (2020) 11:538196. 10.3389/fendo.2020.538196 33071966 PMC7541902

[29] Rimler A Jockers R Lupowitz Z Sampson SR Zisapel N Differential effects of melatonin and its downstream effector PKCα on subcellular localization of RGS proteins. J Pineal Res (2006) 40(2):144–52. 10.1111/j.1600-079X.2005.00290.x 16441551

[30] Gaildrat P Becq F Falcón J First cloning and functional characterization of a melatonin receptor in fish brain: a novel one? J Pineal Res (2002) 32(2):74–84. 10.1034/j.1600-079x.2002.1817.x 12071471

[31] Vaněček J Cellular mechanisms of melatonin action. Physiol Rev (1998) 78(3):687–721. 10.1152/physrev.1998.78.3.687 9674691

[32] Huang H Lee SC Yang XL Modulation by melatonin of glutamatergic synaptic transmission in the carp retina. J Physiol (2005) 569(3):857–71. 10.1113/jphysiol.2005.098798 PMC1464261 16239269

[33] Dubocovich ML Delagrange P Krause DN Sugden D Cardinali DP Olcese J Nomenclature, Classification, and Pharmacology of G Protein-Coupled Melatonin Receptors. Pharmocological Rev (2010) 62(3):343–80. 10.1124/pr.110.002832.343 PMC2964901 20605968

[34] Witt-Enderby PA Bennett J Jarzynka MJ Firestine S Melan MA Melatonin receptors and their regulation: biochemical and structural mechanisms. Life Sci (2003) 72(20):2183–98. 10.1016/S0024-3205(03)00098-5 12628439

[35] Kelberman D Rizzoti K Lovell-Badge R Robinson IC Dattani MT Genetic regulation of pituitary gland development in human and mouse. Endocr Rev (2009) 30(7):790–829. 10.1210/er.2009-0008 19837867 PMC2806371

[36] Pogoda HM Hammerschmidt M Molecular genetics of pituitary development in zebrafish. Semin Cell Dev Biol (2007) 18(4):543–58. 10.1016/j.semcdb.2007.04.004 17560816

[37] Yamamoto K Bloch S Vernier P New perspective on the regionalization of the anterior forebrain in *Osteichthyes*. Dev Growth Differ (2017) 59(4):175–87. 10.1111/dgd.12348 PMC11520958 28470718

[38] Fabian P Tseng K-C Smeeton J Lancman JJ Dong PDS Cerny R Lineage analysis reveals an endodermal contribution to the vertebrate pituitary. Science (80- ) (2020) 370(6515):463–7. 10.1126/science.aba4767 PMC8021009 33093109

[39] Zhu X Gleiberman AS Rosenfeld MG Molecular physiology of pituitary development: Signaling and transcriptional networks. Physiol Rev (2007) 87(3):933–63. 10.1152/physrev.00006.2006 17615393

[40] Weltzien FA Andersson E Andersen Ø Shalchian-Tabrizi K Norberg B The brain-pituitary-gonad axis in male teleosts, with special emphasis on flatfish (Pleuronectiformes). Comp Biochem Physiol - A Mol Integr Physiol (2004) 137(3):447–77. 10.1016/j.cbpb.2003.11.007 15123185

[41] Zohar Y Muñoz-Cueto JA Elizur A Kah O Neuroendocrinology of reproduction in teleost fish. Gen Comp Endocrinol (2010) 165(3):438–55. 10.1016/j.ygcen.2009.04.017 19393655

[42] Kaprara A Huhtaniemi IT The hypothalamus-pituitary-gonad axis: Tales of mice and men. Metabolism (2018) 86:3–17. 10.1016/j.metabol.2017.11.018 29223677

[43] Fontaine R Ciani E Haug TM Hodne K Ager-Wick E Baker DM Gonadotrope plasticity at cellular, population and structural levels: A comparison between fishes and mammals. Gen Comp Endocrinol (2020) 287:113344. 10.1016/j.ygcen.2019.113344 31794734

[44] Fontaine R Royan MR von Krogh K Weltzien F Baker D Direct and indirect effects of sex steroids on gonadotrope cell plasticity in the teleost fish pituitary. Front Endocrinol (Lausanne) (2020) 11:605068. 10.3389/fendo.2020.605068 33365013 PMC7750530

[45] Bittman EL Kaynard AH Olster DH Robinson JE Yellon SM Karsch FJ Pineal melatonin mediates photoperiodic control of pulsatile luteinizing hormone secretion in the ewe. Neuroendocrinology (1985) 40(5):409–18. 10.1159/000124106 3892351

[46] Viguié C Caraty A Locatelli A Malpaux B Regulation of Luteinizing Hormone-Releasing Hormone (LHRH) Secretion by Melatonin in the Ewe.I. Simultaneous Delayed Increase in LHRH and Luteinizing Hormone Pulsatile Secretion1. Biol Reprod (1995) 52(5):1114–20. 10.1095/biolreprod52.5.1114 7626711

[47] El Qandil S Chakir J El Moussaouiti R Oukouchoud R Rami N Benjelloun WA Role of the pineal gland and melatonin in the photoperiodic control of hypothalamic gonadotropin-releasing hormone in the male jerboa (Jaculus orientalis), a desert rodent. Brain Res Bull (2005) 64(5):371–80. 10.1016/j.brainresbull.2004.06.010 15607825

[48] Roy D Angelini NL Fujieda H Brown GM Belsham DD Cyclical regulation of GnRH gene expression in GT1-7 GnRH-secreting neurons by melatonin. Endocrinology (2001) 142(11):4711–20. 10.1210/endo.142.11.8464 11606436

[49] Sato S Yin C Teramoto A Sakuma Y Kato M Sexually dimorphic modulation of GABAA receptor currents by melatonin in rat gonadotropin-releasing hormone neurons. J Physiol Sci (2008) 58(5):317–22. 10.2170/physiolsci.RP006208 18834560

[50] Revel FG Ansel L Klosen P Saboureau M Pévet L Mikkelsen JD Kisspeptin: A key link to seasonal breeding. Rev Endocr Metab Disord (2007) 8(1):57–65. 10.1007/s11154-007-9031-7 17380397

[51] Ansel L Bolborea M Bentsen AH Klosen P Mikkelsen JD Simonneaux V Differential regulation of kiss1 expression by melatonin and gonadal hormones in male and female syrian hamsters. J Biol Rhythms (2010) 25(2):81–91. 10.1177/0748730410361918 20348459

[52] Piekarski DJ Jarjisian SG Perez L Ahmad H Dhawan N Zucker I Effects of pinealectomy and short day lengths on reproduction and neuronal RFRP-3, kisspeptin, and GnRH in female Turkish hamsters. J Biol Rhythms (2014) 29(3):181–91. 10.1177/0748730414532423 PMC4387122 24916391

[53] Li SN Xue HL Zhang Q Xu JH Wang S Chen L Photoperiod regulates the differential expression of KISS-1 and GPR54 in various tissues and sexes of striped hamster. Genet Mol Res (2015) 14(4):13894–905. 10.4238/2015.October.29.10 26535705

[54] Oliveira AC de Andreotti S Sertie RAL Campana AB de Proença ARG Vasconcelos RP Combined treatment with melatonin and insulin improves glycemic control, white adipose tissue metabolism and reproductive axis of diabetic male rats. Life Sci (2018) 199:158–66. 10.1016/j.lfs.2018.02.040 29501522

[55] Mason AO Duffy S Zhao S Ubuka T Bentley GE Tsutsui K Photoperiod and reproductive condition are associated with changes in RFamide-Related Peptide (RFRP) expression in Syrian hamsters (*Mesocricetus auratus*). J Biol Rhythms (2010) 25(3):176–85. 10.1177/0748730410368821 PMC3266107 20484689

[56] Revel FG Saboureau M Pévet P Simonneaux V Mikkelsen JD RFamide-related peptide gene is a melatonin-driven photoperiodic gene. Endocrinology (2008) 149(3):902–12. 10.1210/en.2007-0848 18079200

[57] Ubuka T Inoue K Fukuda Y Mizuno T Ukena K Kriegsfeld LJ Identification, expression, and physiological functions of Siberian hamster gonadotropin-inhibitory hormone. Endocrinology (2012) 153(1):373–85. 10.1210/en.2011-1110 PMC3249677 22045661

[58] Alexiuk NAM Uddin M Vriend J Melatonin increases the in situ activity of tyrosine hydroxylase in the mediobasal hypothalamus of male Syrian hamsters. Life Sci (1996) 59(8):687–94. 10.1016/0024-3205(96)00350-5 8761019

[59] Viguié C Thibault J Thiéry J-CC Tillet Y Malpaux B Characterization of the short day-induced decrease in median eminence tyrosine hydroxylase activity in the ewe: Temporal relationship to the changes in luteinizing hormone and prolactin secretion and short day-like effect of melatonin. Endocrinology (1997) 138(1):499–506. 10.1210/endo.138.1.4865 8977440

[60] Hastings MH Walker AP Roberts AC Herbert J Intra-hypothalamic melatonin blocks photoperiodic responsiveness in the male syrian hamster. Neuroscience (1988) 24(3):987–91. 10.1016/0306-4522(88)90081-4 3380310

[61] Lincoln GA Maeda KI Effects of placing micro-implants of melatonin in the mediobasal hypothalamus and preoptic area on the secretion of prolactin and β-endorphin in rams. J Endocrinol (1992) 134(3):437–48. 10.1677/joe.0.1340437 1402551

[62] Malpaux B Daveau A Maurice F Gayrard V Thiery J-C Short-Day Effects of Melatonin on Luteinizing Hormone Secretion in the Ewe: Evidence for Central Sites of Action in the Mediobasal Hypothalamus1. Biol Reprod (1993) 48(4):752–60. 10.1095/biolreprod48.4.752 8485239

[63] Maywood ES Lesions of the iodomelatonin-binding sites of the mediobasal hypothalamus spare the lactotropic, but block the gonadotropic response of male Syrian hamsters to short photoperiod and to melatonin. Endocrinology (1995) 136(1):144–53. 10.1210/en.136.1.144 7828525

[64] Bae HH Mangels RA Cho BS Dark J Yellon SM Zucker I Ventromedial hypothalamic mediation of photoperiodic gonadal responses in male Syrian hamsters. J Biol Rhythms (1999) 14(5):391–401. 10.1177/074873099129000795 10511006

[65] Lewis D Freeman DA Dark J Wynne-Edwards KE Zucker I Photoperiodic control of oestrous cycles in Syrian hamsters: Mediation by the mediobasal hypothalamus. J Neuroendocrinol (2002) 14(4):294–9. 10.1046/j.1365-2826.2002.00779.x 11963826

[66] Vivid D Bentley GE Seasonal Reproduction in Vertebrates: Melatonin Synthesis, Binding, and Functionality Using Tinbergen’s Four Questions. Molecules (2018) 23(3):652. 10.3390/molecules23030652 PMC6017951 29534047

[67] Dardente H Wood S Ebling F Sáenz de Miera C An integrative view of mammalian seasonal neuroendocrinology. J Neuroendocrinol (2019) 31(5):e12729. 10.1111/jne.12729 31059174

[68] Ciani E Fontaine R Maugars G Nourizadeh-Lillabadi R Andersson E Bogerd J Gnrh receptor gnrhr2bbα is expressed exclusively in lhb-expressing cells in Atlantic salmon male parr. Gen Comp Endocrinol (2020) 285:113293. 10.1016/j.ygcen.2019.113293 31580881

[69] Stewart AJ Katz AA Millar RP Morgan K Retention and silencing of prepro-GnRH-II and type II GnRH receptor genes in mammals. Neuroendocrinology (2009) 90(4):416–32. 10.1159/000233303 19657181

[70] Roy D Belsham DD Melatonin receptor activation regulates GnRH gene expression and secretion in GT1-7 GnRH neurons. Signal transduction mechanisms. J Biol Chem (2002) 277(1):251–8. 10.1074/jbc.M108890200 11684691

[71] Ishii H Tanaka N Kobayashi M Kato M Sakuma Y Gene structures, biochemical characterization and distribution of rat melatonin receptors. J Physiol Sci (2009) 59(1):37–47. 10.1007/s12576-008-0003-9 19340560 PMC10717452

[72] Kriegsfeld LJ Mei DF Bentley GE Ubuka T Mason AO Inoue K Identification and characterization of a gonadotropin-inhibitory system in the brains of mammals. Proc Natl Acad Sci USA (2006) 103(7):2410–5. 10.1073/pnas.0511003103 PMC1413747 16467147

[73] Ancel C Bentsen AH Sébert ME Tena-Sempere M Mikkelsen JD Simonneaux V Stimulatory effect of RFRP-3 on the gonadotrophic axis in the male Syrian hamster: The exception proves the rule. Endocrinology (2012) 153(3):1352–63. 10.1210/en.2011-1622 22275511

[74] Dardente H Birnie M Lincoln GA Hazlerigg DG RFamide-Related peptide and its cognate receptor in the sheep: cDNA cloning, mRNA distribution in the hypothalamus and the effect of photoperiod. J Neuroendocrinol (2008) 20(11):1252–9. 10.1111/j.1365-2826.2008.01784.x 18752651

[75] Smith JT Coolen LM Kriegsfeld LJ Sari IP Jaafarzadehshirazi MR Maltby M Variation in kisspeptin and RFamide-related peptide (RFRP) expression and terminal connections to gonadotropin-releasing hormone neurons in the brain: A novel medium for seasonal breeding in the sheep. Endocrinology (2008) 149(11):5770–82. 10.1210/en.2008-0581 PMC2584593 18617612

[76] Harbid AA Mcleod BJ Caraty A Anderson GM Seasonal changes in RFamide-related peptide-3 neurons in the hypothalamus of a seasonally breeding marsupial species, the brushtail possum (*Trichosurus vulpecula*). J Comp Neurol (2013) 521(13):3030–41. 10.1002/cne.23328 23504980

[77] Kriegsfeld LJ Ubuka T Bentley GE Tsutsui K Seasonal control of gonadotropin-inhibitory hormone (GnIH) in birds and mammals. Front Neuroendocrinol (2015) 37:65–75. 10.1016/j.yfrne.2014.12.001 25511257 PMC4405439

[78] Simonneaux V A Kiss to drive rhythms in reproduction. Eur J Neurosci (2020) 51(1):509–30. 10.1111/ejn.14287 30472752

[79] Rasri-Klosen K Simonneaux V Klosen P Differential response patterns of kisspeptin and RFamide-related peptide to photoperiod and sex steroid feedback in the Djungarian hamster (*Phodopus sungorus*). J Neuroendocrinol (2017) 29(9). 10.1111/jne.12529 28834570

[80] Gingerich S Wang X Lee PKP Dhillon SS Chalmers JA Koletar MM The generation of an array of clonal, immortalized cell models from the rat hypothalamus: analysis of melatonin effects on kisspeptin and gonadotropin-inhibitory hormone neurons. Neuroscience (2009) 162(4):1134–40. 10.1016/j.neuroscience.2009.05.026 19463905

[81] Li Q Rao A Pereira A Clarke IJ Smith JT Kisspeptin Cells in the Ovine Arcuate Nucleus Express Prolactin Receptor but not Melatonin Receptor. J Neuroendocrinol (2011) 23(10):871–82. 10.1111/j.1365-2826.2011.02195.x 21793946

[82] Dufour S Sebert ME Weltzien FA Rousseau K Pasqualini C Neuroendocrine control by dopamine of teleost reproduction. J Fish Biol (2010) 76(1):129–60. 10.1111/j.1095-8649.2009.02499.x 20738703

[83] Angelousi A Margioris AN Tsatsanis C ACTH Action on the Adrenal. In: Endotext. South Dartmouth (MA): MDText.com, Inc. (2020). http://www.ncbi.nlm.nih.gov/pubmed/25905342

[84] Aoyama H Mori N Mori W Anti-glucocorticoid effects of melatonin on adult rats. Pathol Int (1987) 37(6):1143–8. 10.1111/j.1440-1827.1987.tb00431.x 3661196

[85] Pierpaoli W Maestroni GJM Melatonin: a principal neuroimmunoregulatory and anti-stress hormone: its anti-aging effects. Immunol Lett (1987) 16(3-4):355–61. 10.1016/0165-2478(87)90169-6 3327818

[86] Konakchieva R Mitev Y Almeida OF Patchev VK Chronic melatonin treatment and the hypothalamo-pituitary-adrenal axis in the rat: Attenuation of the secretory response to stress and effects on hypothalamic neuropeptide content and release. Biol Cell (1997) 89(9):587–96. 10.1111/j.1768-322x.1997.tb01036.x 9673011

[87] Konakchieva R Mitev Y Almeida OFX Patchev VK Chronic melatonin treatment counteracts glucocorticoid-induced dysregulation of the hypothalamic-pituitary-adrenal axis in the rat. Neuroendocrinology (1998) 67(3):171–80. 10.1159/000054312 9630434

[88] Fischer S Smolnik R Herms M Born J Fehm HL Melatonin Acutely Improves the Neuroendocrine Architecture of Sleep in Blind Individuals. J Clin Endocrinol Metab (2003) 88(11):5315–20. 10.1210/jc.2003-030540 14602767

[89] Wu YH Zhou JN Balesar R Unmehopa U Bao A Jockers B Distribution of MT1 melatonin receptor immunoreactivity in the human hypothalamus and pituitary gland: Colocalization of MT1 with vasopressin, oxytocin, and corticotropin-releasing hormone. J Comp Neurol (2006) 499(6):897–910. 10.1002/cne.21152 17072839

[90] Bernard V Young J Binart N Prolactin — a pleiotropic factor in health and disease. Nat Rev Endocrinol (2019) 15(6):356–65. 10.1038/s41574-019-0194-6 30899100

[91] Pellicer-Rubio MT Boissard K Forgerit Y Pougnard JL Bonné JL Leboeuf B Evaluation of hormone-free protocols based on the “male effect” for artificial insemination in lactating goats during seasonal anestrus. Theriogenology (2016) 85(5):960–9. 10.1016/j.theriogenology.2015.11.005 26707385

[92] Freeman ME Kanyicska B Lerant A Nagy G Prolactin: Structure, function, and regulation of secretion. Physiol Rev (2000) 80(4):1523–631. 10.1152/physrev.2000.80.4.1523 11015620

[93] Molik E Misztal T Romanowicz K Zieba D Short-day and melatonin effects on milking parameters, prolactin profiles and growth-hormone secretion in lactating sheep. Small Rumin Res (2013) 109(2-3):182–7. 10.1016/j.smallrumres.2012.10.006

[94] Ponchon B Lacasse P Ollier S Zhao X Effects of photoperiod modulation and melatonin feeding around drying-off on bovine mammary gland involution. J Dairy Sci (2017) 100(10):8496–506. 10.3168/jds.2016-12272 28755938

[95] Sanchez-Barcelo EJ Mediavilla MD Zinn SA Buchanan BA Chapin LT Tucker HA Melatonin suppression of mammary growth in heifers. Biol Reprod (1991) 44(5):875–9. 10.1095/biolreprod44.5.875 1868145

[96] Dahl GE Buchanan BA Tucker HA Photoperiodic effects on dairy cattle: A review. J Dairy Sci (2000) 83(4):885–93. 10.3168/jds.S0022-0302(00)74952-6 10791806

[97] Lincoln GA Tortonese DJ Does melatonin act on dopaminergic pathways in the mediobasal hypothalamus to mediate effects of photoperiod on prolactin secretion in the ram? Neuroendocrinology (1995) 62(5):425–33. 10.1159/000127032 8559274

[98] Lincoln GA Clarke IJ Evidence that Melatonin Acts in the Pituitary Gland through a Dopamine-independent Mechanism to Mediate Effects of Daylength on the Secretion of Prolactin in the Ram. J Neuroendocrinol (1995) 7(8):637–43. 10.1111/j.1365-2826.1995.tb00802.x 8704738

[99] Ranke MB Wit JM Growth hormone-past, present and future. Nat Rev Endocrinol (2018) 14(5):285–300. 10.1038/nrendo.2018.22 29546874

[100] Sarapura VD Samuel MH Thyroid-Stimulating Hormone. In: The Pituitary: Fourth Edition. New York (USA): Elsevier Inc (2017). p. 163–201. 10.1016/B978-0-12-804169-7.00006-4

[101] Gross DS The mammalian hypophysial pars tuberalis: A comparative immunocytochemical study. Gen Comp Endocrinol (1984) 56(2):283–98. 10.1016/0016-6480(84)90043-1 6510690

[102] Sakai T Inoue K Kurosumi K Light and Electron Microscopic Immunocytochemistry of TSH-like Cells Occurring in the Pars tuberalis of the Adult Male Rat Pituitary. Arch Histol Cytol (1992) 55(2):151–7. 10.1679/aohc.55.151 1497945

[103] Wittkowski W Bergmann M Hoffmann K Pera F Photoperiod-dependent changes in TSH-like immunoreactivity of cells in the hypophysial pars tuberalis of the Djungarian hamster, Phodopus sungorus. Cell Tissue Res (1988) 251(1):183–7. 10.1007/BF00215463 3342436

[104] Joseph-Bravo P Jaimes-Hoy L Uribe RM Charli JL TRH, the first hypophysiotropic releasing hormone isolated: Control of the pituitary-thyroid axis. J Endocrinol (2015) 226(2):T85–T100. 10.1530/JOE-15-0124 26101376

[105] Bockmann J Böckers TM Winter C Wittkowski W Winterhoff H Deufel T Thyrotropin Expression in Hypophyseal Pars Tuberalis-Specific Cells is 3,5,3′-Triiodothyronine, Thyrotropin-Releasing Hormone, and Pit-1 Independent*. Endocrinology (1997) 138(3):1019–28. 10.1210/endo.138.3.5007 9048604

[106] Herrera-Pérez P Del Carmen Rendón M Besseau L Sauzet S Falcón J Muñoz-Cueto JA Melatonin receptors in the brain of the European sea bass: An in situ hybridization and autoradiographic study. J Comp Neurol (2010) 518(17):3495–511. 10.1002/cne.22408 20589910

[107] Martinoli MG Williams LM Kah O Titchener LT Pelletier G Distribution of central melatonin binding sites in the goldfish (Carassius auratus). Mol Cell Neurosci (1991) 2(1):78–85. 10.1016/1044-7431(91)90042-m 19912786

[108] Mazurais D Brierley I Anglade I Drew J Randall C Bromage N Central melatonin receptors in the rainbow trout: Comparative distribution of ligand binding and gene expression. J Comp Neurol (1999) 409(2):313–24. 10.1002/(SICI)1096-9861(19990628)409:2<313::AID-CNE11>3.0.CO;2-1 10379923

[109] Iversen M Myhr AI Wargelius A Approaches for delaying sexual maturation in salmon and their possible ecological and ethical implications. J Appl Aquac (2016) 28(4):330–69. 10.1080/10454438.2016.1212756

[110] Burgerhout E Lokman PM van den Thillart GEEJM Dirks RP The time-keeping hormone melatonin: a possible key cue for puberty in freshwater eels (*Anguilla spp*.). Rev Fish Biol Fish (2019) 29(1):1–21. 10.1007/s11160-018-9540-3

[111] Carnevali O Gioacchini G Maradonna F Olivotto I Migliarini B Melatonin induces follicle maturation in *danio rerio*. Hansen IA, ed. PLoS One (2011) 6(5):e19978. 10.1371/journal.pone.0019978 21647435 PMC3102064

[112] Kim JH Park JW Jin YH Kim DJ Kwon JY Effect of melatonin on GnIH precursor gene expression in Nile tilapia, Oreochromis niloticus. Biol Rhythm Res (2018) 49(2):303–13. 10.1080/09291016.2017.1357336

[113] Amano M Iigo M Ikuta K Kitamura S Okuzawa K Yamada H Disturbance of plasma melatonin profile by high dose melatonin administration inhibits testicular maturation of precocious male masu salmon. Zoolog Sci (2004) 21(1):79–85. 10.2108/0289-0003(2004)21[79:DOPMPB]2.0.CO;2 14745107

[114] Servili A Herrera-Pérez P Rendón M del C Muñoz-Cueto JA Melatonin inhibits GnRH-1, GnRH-3 and GnRH receptor expression in the brain of the European sea bass, *Dicentrarchus labrax*. Int J Mol Sci (2013) 14(4):7603–16. 10.3390/ijms14047603 PMC3645706 23567273

[115] Alvarado MV Carrillo M Felip A Melatonin-induced changes in kiss/gnrh gene expression patterns in the brain of male sea bass during spermatogenesis. Comp Biochem Physiol -Part A Mol Integr Physiol (2015) 185:69–79. 10.1016/j.cbpa.2015.03.010 25810361

[116] Sébert ME Legros C Weltzien FA Malpaux B Chemineau P Dufour S Melatonin activates brain dopaminergic systems in the eel with an inhibitory impact on reproductive function. J Neuroendocrinol (2008) 20(7):917–29. 10.1111/j.1365-2826.2008.01744.x 18445127

[117] Yumnamcha T Khan ZA Rajiv C Devi SD Mondal G Sanjita Devi H Interaction of melatonin and gonadotropin-inhibitory hormone on the zebrafish brain-pituitary-reproductive axis. Mol Reprod Dev (2017) 84(5):389–400. 10.1002/mrd.22795 28295807

[118] Popek W Łuszczek-Trojnar E Dra̧g-Kozak E Fortuna-Wrońska D Epler P Effect of the pineal gland and melatonin on dopamine release from perifused hypothalamus of mature female carp during spawning and winter regression. Acta Ichthyol Piscat (2005) 35(2):65–71. 10.3750/AIP2005.35.2.01

[119] Popek W Łuszczek-Trojnar E Dra̧g-Kozak E Rza̧sa J Epler P Effect of melatonin on dopamine secretion in the hypothalamus of mature female common carp, Cyprinus carpio L. Acta Ichthyol Piscat (2006) 36(2):135–41. 10.3750/AIP2006.36.2.07

[120] Chaube R Joy KP Effects of altered photoperiod and temperature, serotonin-affecting drugs, and melatonin on brain tyrosine hydroxylase activity in female catfish, Heteropneustes fossilis: A study correlating ovarian activity changes. J Exp Zool (2002) 293(6):585–93. 10.1002/jez.10185 12410607

[121] Senthilkumaran B Joy KP Effects of melatonin, p-chlorophenylalanine, and α-methylparatyrosine on plasma gonadotropin level and ovarian activity in the catfish, Heteropneustes fossilis: A study correlating changes in hypothalamic monoamines. Fish Physiol Biochem (1995) 14(6):471–80. 10.1007/BF00004347 24197643

[122] Hernández-Rauda R Miguez JM Ruibal C Aldegunde M Effects of melatonin on dopamine metabolism in the hypothalamus and the pituitary of the rainbow trout, Oncorhynchus mykiss. J Exp Zool (2000) 287(6):440–4. 10.1002/1097-010X(20001101)287:6<440::AID-JEZ5>3.0.CO;2-S 11074456

[123] Levavi-Sivan B Bogerd J Mañanós EL Gómez A Lareyre JJ Perspectives on fish gonadotropins and their receptors. Gen Comp Endocrinol (2010) 165(3):412–37. 10.1016/j.ygcen.2009.07.019 19686749

[124] Schulz RW de França LR Lareyre JJ LeGac F Chiarini-Garcia H Nobrega RH Spermatogenesis in fish. Gen Comp Endocrinol (2010) 165(3):390–411. 10.1016/j.ygcen.2009.02.013 19348807

[125] Campos-Mendoza A McAndrew BJ Coward K Bromage N Reproductive response of Nile tilapia (Oreochromis niloticus) to photoperiodic manipulation; Effects on spawning periodicity, fecundity and egg size. Aquaculture (2004) 231(1-4):299–314. 10.1016/j.aquaculture.2003.10.023

[126] Ridha MT Cruz EM Effect of light intensity and photoperiod on nile tilapia Oreochromis niloticus L. seed production. Aquac Res (2000) 31(7):609–17. 10.1046/j.1365-2109.2000.00481.x

[127] Bayarri MJ Rodríguez L Zanuy S Madrid JA Sánchez-Vázquez FJ Kagawa H Effect of photoperiod manipulation on the daily rhythms of melatonin and reproductive hormones in caged European sea bass (*Dicentrarchus labrax*). Gen Comp Endocrinol (2004) 136(1):72–81. 10.1016/j.ygcen.2003.12.004 14980798

[128] Servili A Lethimonier C Lareyre JJ López-Olmeda JF Sánchez-Vázquez FJ Kah O The highly conserved gonadotropin-releasing hormone-2 form acts as a melatonin-releasing factor in the pineal of a teleost fish, the European sea bass *Dicentrarchus labrax*. Endocrinology (2010) 151(5):2265–75. 10.1210/en.2009-1207 20215565

[129] Amano M Iigo M Ikuta K Kitamura S Yamada H Yamamori K Roles of melatonin in gonadal maturation of underyearling precocious male masu salmon. Gen Comp Endocrinol (2000) 120(2):190–7. 10.1006/gcen.2000.7547 11078630

[130] Kim JH Park JW Kwon JY Effects of exogenous melatonin on the reproductive activities of Nile tilapia, Oreochromis niloticus. Biol Rhythm Res (2018) 49(3):392–404. 10.1080/09291016.2017.1366715

[131] Tsutsui K Ubuka T Yin H Osugi T Ukena K Bentley GE Discovery of gonadotropin-inhibitory hormone in a domesticated bird, its mode of action and functional significance. J Ornithol (2007) 148(Suppl 2):S515–S20. 10.1007/s10336-007-0225-2

[132] Choi YJ Habibi HR Choi CY Profiles of gonadotropin-inhibitory hormone and melatonin during the sex change and maturation of cinnamon clownfish, Amphiprion melanopus. Biochem Biophys Res Commun (2016) 475(2):189–93. 10.1016/j.bbrc.2016.05.073 27208779

[133] Somoza GM Mechaly AS Trudeau VL Kisspeptin and GnRH interactions in the reproductive brain of teleosts. Gen Comp Endocrinol (2020) 298:113568. 10.1016/j.ygcen.2020.113568 32710898

[134] Pasquier J Kamech N Lafont AG Vaudry H Rousseau K Dufour S Molecular evolution of GPCRs: Kisspeptin/kisspeptin receptors. J Mol Endocrinol (2014) 52(3):101–17. 10.1530/JME-13-0224 24577719

[135] Escobar S Servili A Espigares F Gueguen MM Brocal I Felip A Expression of Kisspeptins and Kiss Receptors Suggests a Large Range of Functions for Kisspeptin Systems in the Brain of the European Sea Bass. PLoS One (2013) 8(7):e70177. 10.1371/journal.pone.0070177 23894610 PMC3720930

[136] Takemura A Uchimura M Shibata Y Dopaminergic activity in the brain of a tropical wrasse in response to changes in light and hydrostatic pressure. Gen Comp Endocrinol (2010) 166(3):513–9. 10.1016/j.ygcen.2010.01.001 20064517

[137] Ekström P Vaněček J Localization of 2-[125I]lodomelatonin binding sites in the brain of the atlantic salmon, salmo salar L. Neuroendocrinology (1992) 55(5):529–37. 10.1159/000126166 1316562

[138] Iigo M Kobayashi M Ohtani-Kaneko R Hara M Hattori A Suzuki T Characteristics, day-night changes, subcellular distribution and localization of melatonin binding sites in the goldfish brain. Brain Res (1994) 644(2):213–20. 10.1016/0006-8993(94)91682-9 8050032

[139] Iigo M Sánchez-Vázquez FJ Hara M Ohtani-Kaneko R Hirata K Shinohara H Characterization, guanosine 5’-O-(3-thiotriphosphate) modulation, daily variation, and localization of melatonin-binding sites in the catfish (Silurus asotus) brain. Gen Comp Endocrinol (1997) 108(1):45–55. 10.1006/gcen.1997.6940 9378273

[140] Morgan PJ Webster CA Mercer JG Ross AW Hazlerigg DG MacLean A The ovine pars tuberalis secretes a factor(s) that regulates gene expression in both lactotropic and nonlactotropic pituitary cells. Endocrinology (1996) 137(9):4018–26. 10.1210/endo.137.9.8756579 8756579

[141] Hazlerigg DG Gonzalez-Brito A Lawson W Hastings MH Morgan PJ Prolonged exposure to melatonin leads to time-dependent sensitization of adenylate cyclase and down-regulates melatonin receptors in pars tuberalis cells from ovine pituitary. Endocrinology (1993) 132(1):285–92. 10.1210/endo.132.1.7678217 7678217

[142] Barrett P Davidson G Hazlerigg DG Morris MA Ross AW Morgan PJ Mel 1a melatonin receptor expression is regulated by protein kinase C and an additional pathway addressed by the protein kinase C inhibitor Ro 31-8220 in ovine pars tuberalis cells. Endocrinology (1998) 139(1):163–71. 10.1210/endo.139.1.5699 9421411

[143] Fustin JM Dardente H Wagner GC Carter DA Johnston JD Lincoln GA Egr1 involvement in evening gene regulation by melatonin. FASEB J (2009) 23(3):764–73. 10.1096/fj.08-121467 19019852

[144] Martin JE Klein DC Melatonin inhibition of the neonatal pituitary response to luteinizing hormone-releasing factor. Science (80- ) (1976) 191(4224):301–2. 10.1126/science.1108199 1108199

[145] Martin JE Sattler C Selectivity of melatonin pituitary inhibition for luteinizing hormone-releasing hormone. Neuroendocrinology (1982) 34(2):112–6. 10.1159/000123287 6122168

[146] Vaněček J Klein DC Melatonin inhibition of GnRH-induced LH release from neonatal rat gonadotroph: Involvement of Ca2+ not cAMP. Am J Physiol - Endocrinol Metab (1995) 269(1 32-1). 10.1152/ajpendo.1995.269.1.e85 7631782

[147] Sumova A Vaněček J Melatonin Inhibits GnRH-Induced Increase of cFOS Immunoreactivity in Neonatal Rat Pituitary. J Neuroendocrinol (1997) 9(2):135–9. 10.1046/j.1365-2826.1997.d01-1076.x 9041367

[148] Rivest RW Jaconi MEE Gruaz N Sizonenko PC Aubert ML Short-term and long-term effects of melatonin on GnRH-stimulated gonadotropin secretion in pituitaries of sexually maturing rats. Neuroendocrinology (1987) 46(5):379–86. 10.1159/000124848 3124012

[149] Nakazawa K Marubayashi U McCann SM Mediation of the short-loop negative feedback of luteinizing hormone (LH) on LH-releasing hormone release by melatonin-induced inhibition of LH release from the pars tuberalis. Proc Natl Acad Sci USA (1991) 88(17):7576–9. 10.1073/pnas.88.17.7576 PMC52344 1881898

[150] Griffiths D Bjoro T Gautvik K Haug E Melatonin reduces the production and secretion of prolactin and growth hormone from rat pituitary cells in culture. Acta Physiol Scand (1987) 131(1):43–9. 10.1111/j.1748-1716.1987.tb08203.x 2823532

[151] Ogura-Ochi K Fujisawa S Iwata N Komatsubara M Nishiyama Y Tsukamoto-Yamauchi N Regulatory role of melatonin and BMP-4 in prolactin production by rat pituitary lactotrope GH3 cells. Peptides (2017) 94:19–24. 10.1016/j.peptides.2017.06.001 28627372

[152] Ibáñez-Costa A Córdoba-Chacón J Gahete MD Kineman RD Castaño JP Luque RM Melatonin regulates somatotrope and lactotrope function through common and distinct signaling pathways in cultured primary pituitary cells from female primates. Endocrinology (2015) 156(3):1100–10. 10.1210/en.2014-1819 PMC4330310 25545385

[153] Tsukamoto N Otsuka F Ogura-Ochi K Inagaki K Nakamura E Toma K Melatonin receptor activation suppresses adrenocorticotropin production via BMP-4 action by pituitary AtT20 cells. Mol Cell Endocrinol (2013) 375(1-2):1–9. 10.1016/j.mce.2013.05.010 23701823

[154] Reppert SM Weaver DR Ebisawa T Cloning and characterization of a mammalian melatonin receptor that mediates reproductive and circadian responses. Neuron (1994) 13(5):1177–85. 10.1016/0896-6273(94)90055-8 7946354

[155] Weaver DR Rivkees SA Reppert SM Localization and characterization of melatonin receptors in rodent brain by in vitro autoradiography. J Neurosci (1989) 9(7):2581–90. 10.1523/jneurosci.09-07-02581.1989 PMC6569774 2545841

[156] Schuster C Gauer F Malan A Recio J Pévet P Masson-Pévet M The circadian clock, light/dark cycle and melatonin are differentially involved in the expression of daily and photoperiodic variations in mt1 melatonin receptors in the Siberian and Syrian hamsters. Neuroendocrinology (2001) 74(1):55–68. 10.1159/000054670 11435758

[157] Klosen P Bienvenu C Demarteau O Dardente H Guerrero H Pévet P The mt1 melatonin receptor and RORβ receptor are co-localized in specific TSH-immunoreactive cells in the pars tuberalis of the rat pituitary. J Histochem Cytochem (2002) 50(12):1647–57. 10.1177/002215540205001209 12486087

[158] Williams LM Morgan PJ Demonstration of melatonin-binding sites on the pars tuberalis of the rat. J Endocrinol (1988) 119(1):R1–3. 10.1677/joe.0.119R001 2848087

[159] Williams LM Hannah LT Kyle CE Adam CL Central melatonin receptors in red deer (*Cervus elaphus*). Gen Comp Endocrinol (1996) 104(1):1–6. 10.1006/gcen.1996.0134 8921349

[160] Weaver DR Reppert SM Melatonin receptors are present in the ferret pars tuberalis and pars distalis, but not in brain. Endocrinology (1990) 127(5):2607–9. 10.1210/endo-127-5-2607 2171920

[161] Weaver DR Stehle JH Stopa EG Reppert SM Melatonin receptors in human hypothalamus and pituitary: Implications for circadian and reproductive responses to melatonin. J Clin Endocrinol Metab (1993) 76(2):295–301. 10.1210/jcem.76.2.8381796 8381796

[162] Barrett P MacLean A Davidson G Morgan PJ Regulation of the Mel 1a melatonin receptor mRNA and protein levels in the ovine pars tuberalis: Evidence for a cyclic adenosine 3’,5’- monophosphate-independent Mel 1a receptor coupling and an autoregulatory mechanism of expression. Mol Endocrinol (1996) 10(7):892–902. 10.1210/me.10.7.892 8813729

[163] Dardente H Klosen P Pévet P Masson-Pévet M MT1 melatonin receptor mRNA expressing cells in the pars tuberalis of the European hamster: Effect of photoperiod. J Neuroendocrinol (2003) 15(8):778–86. 10.1046/j.1365-2826.2003.01060.x 12834439

[164] Gauer F Masson-Pévet M Pévet P Seasonal regulation of melatonin receptors in rodent pars tuberalis: correlation with reproductive state. J Neural Transm (1994) 96(3):187–95. 10.1007/BF01294786 7826570

[165] Tamarkin L Westrom WK Hamill AI Goldman BD Effect of melatonin on the reproductive systems of male and female syrian hamsters: A diurnal rhythm in sensitivity to melatonin12. Endocrinology (1976) 99(6):1534–41. 10.1210/endo-99-6-1534 1033827

[166] Skene DJ Masson-Pevet M Pevet P Seasonal changes in melatonin binding sites in the pars tuberalis of male european hamsters and the effect of testosterone manipulation. Endocrinology (1993) 132(4):1682–6. 10.1210/endo.132.4.8462468 8462468

[167] Gauer F Masson-Pévet M Saboureau M George D Pévet P Differential Seasonal Regulation of Melatonin Receptor Density in the Pars Tuberalis and the Suprachiasmatic Nuclei: A Study in the Hedgehog (*Erinaceus europaeus*, L.). J Neuroendocrinol (1993) 5(6):685–90. 10.1111/j.1365-2826.1993.tb00540.x 8680442

[168] Messager S Caillol M George D Martinet L Seasonal Variation Of Melatonin Binding Sites In The Pars Tuberalis of the Male Mink (*Mustela vison*). J Neuroendocrinol (1997) 9(7):523–8. 10.1046/j.1365-2826.1997.d01-1122.x 15305570

[169] Johnston JD Messager S Barrett P Hazlerigg DG Melatonin action in the pituitary: Neuroendocrine synchronizer and developmental modulator? J Neuroendocrinol (2003) 15(4):405–8. 10.1046/j.1365-2826.2003.00972.x 12622841

[170] Bae SE Wright IK Wyse C Samson-Desvignes N Le Blanc P Laroche S Regulation of pituitary MT1 melatonin receptor expression by gonadotrophin-releasing hormone (GnRH) and early growth response factor-1 (Egr-1): In vivo and in vitro studies. PLoS One (2014) 9(3)e90056. 10.1371/journal.pone.0090056 PMC3962332 24658054

[171] Bittman EL Weaver DR The Distribution of Melatonin Binding Sites in Neuroendocrine Tissues of the Ewe1. Biol Reprod (1990) 43(6):986–93. 10.1095/biolreprod43.6.986 2291931

[172] Heiliwell RJA Williams LM Melatonin Binding Sites in the Ovine Brain and Pituitary: Characterization During the Oestrous Cycle. J Neuroendocrinol (1992) 4(3):287–94. 10.1111/j.1365-2826.1992.tb00170.x 21554608

[173] Ono H Hoshino Y Yasuo S Watanabe M Nakane Y Murai A Involvement of thyrotropin in photoperiodic signal transduction in mice. Proc Natl Acad Sci USA (2008) 105(47):18238–42. 10.1073/pnas.0808952105 PMC2587639 19015516

[174] Yasuo S Yoshimura T Ebihara S Korf HW Melatonin transmits photoperiodic signals through the MT1 melatonin receptor. J Neurosci (2009) 29(9):2885–9. 10.1523/JNEUROSCI.0145-09.2009 PMC6666200 19261884

[175] Ikegami K Liao XH Hoshino Y Ono H Ota W Ito Y Tissue-specific posttranslational modification allows functional targeting of thyrotropin. Cell Rep (2014) 9(3):801–9. 10.1016/j.celrep.2014.10.006 PMC4251493 25437536

[176] Dardente H Wyse CA Birnie MJ Dupré SM Loudon ASI Lincoln GA A molecular switch for photoperiod responsiveness in mammals. Curr Biol (2010) 20(24):2193–8. 10.1016/j.cub.2010.10.048 21129971

[177] Wood S Loudon A Clocks for all seasons: unwinding the roles and mechanisms of circadian and interval timers in the hypothalamus and pituitary. J Endocrinol (2014) 222(2):R39–59. 10.1530/JOE-14-0141 PMC4104039 24891434

[178] Watanabe M Yasuo S Watanabe T Yamamura T Nakao N Ebihara S Photoperiodic regulation of type 2 deiodinase gene in djungarian hamster: Possible homologies between avian and mammalian photoperiodic regulation of reproduction. Endocrinology (2004) 145(4):1546–9. 10.1210/en.2003-1593 14726436

[179] Korf HW Signaling pathways to and from the hypophysial pars tuberalis, an important center for the control of seasonal rhythms. Gen Comp Endocrinol (2018) 258:236–43. 10.1016/j.ygcen.2017.05.011 28511899

[180] Dupré SM Miedzinska K Duval CV Yu L Goodman RL Lincoln GA Identification of Eya3 and TAC1 as Long-Day Signals in the Sheep Pituitary. Curr Biol (2010) 20(9):829–35. 10.1016/j.cub.2010.02.066 PMC2887296 20434341

[181] Yasuo S Korf HW The hypophysial pars tuberalis transduces photoperiodic signals via multiple pathways and messenger molecules. Gen Comp Endocrinol (2011) 172(1):15–22. 10.1016/j.ygcen.2010.11.006 21078321

[182] Wood S Loudon A The pars tuberalis: The site of the circannual clock in mammals? Gen Comp Endocrinol (2018) 258:222–35. 10.1016/j.ygcen.2017.06.029 28669798

[183] Gauer F Masson-Pévet M Pévet P Melatonin receptor density is regulated in rats pars tuberalis and suprachiasmatic nuclei by melatonin itself. Brain Res (1993) 602(1):153–6. 10.1016/0006-8993(93)90256-M 8383569

[184] Morgan PJ Williams LM The pars tuberalis of the pituitary: A gateway for neuroendocrine output. Rev Reprod (1996) 1(3):153–61. 10.1530/ror.0.0010153 9414453

[185] Dardente H Does a melatonin-dependent circadian oscillator in the pars tuberalis drive prolactin seasonal rhythmicity? J Neuroendocrinol (2007) 19(8):657–66. 10.1111/j.1365-2826.2007.01564.x 17620107

[186] Messager S Ross AW Barrett P Morgan PJ Decoding photoperiodic time through Per1 and ICER gene amplitude. Proc Natl Acad Sci USA (1999) 96(17):9938–43. 10.1073/pnas.96.17.9938 PMC22314 10449798

[187] Poirel VJ Boggio V Dardente H Pevet P Masson-Pevet M Gauer F Contrary to other non-photic cues, acute melatonin injection does not induce immediate changes of clock gene mRNA expression in the rat suprachiasmatic nuclei. Neuroscience (2003) 120(3):745–55. 10.1016/S0306-4522(03)00344-0 12895514

[188] Johnston JD Tournier BB Andersson H Masson-Pévet M Lincoln GA Hazlerigg DG Multiple effects of melatonin on rhythmic clock gene expression in the mammalian pars tuberalis. Endocrinology (2006) 147(2):959–65. 10.1210/en.2005-1100 16269454

[189] Pelíšek V Vaněček J Different effects of melatonin pretreatment on cAMP and LH responses of the neonatal rat pituitary cells. J Pineal Res (2000) 28(4):234–41. 10.1034/j.1600-079X.2000.280406.x 10831159

[190] Balik A Kretschmannová K Mazna P Svobodová I Zemková H Melatonin action in neonatal gonadotrophs. Physiol Res (2004) 53 Suppl 1(SUPPL. 1):S153–66 http://www.biomed.cas.cz/physiolres. 15119946

[191] Zemkova H Vaněček J Dual effect of melatonin on gonadotropin-releasing-hormone-induced Ca2+ signaling in neonatal rat gonadotropes. Neuroendocrinology (2001) 74(4):262–9. 10.1159/000054693 11598382

[192] Zemková H Vaněček J Inhibitory effect of melatonin on gonadotropin-releasing hormone-induced Ca2+ Oscillations in pituitary cells of newborn rats. Neuroendocrinology (1997) 65(4):276–83. 10.1159/000127185 9142999

[193] Deery DJ Effect of catecholamines and synthetic mammalian hypothalamic hormones on the adenylyl cyclase activity of the pituitary of the teleost, Carassius auratus. Gen Comp Endocrinol (1975) 25(4):395–9. 10.1016/0016-6480(75)90149-5 805742

[194] Somoza GM Peter RE Effects of serotonin on gonadotropin and growth hormone release from in vitro perifused goldfish pituitary fragments. Gen Comp Endocrinol (1991) 82(1):103–10. 10.1016/0016-6480(91)90301-l 1874380

[195] Khan IA Thomas P Melatonin influences gonadotropin II secretion in the Atlantic croaker (*Micropogonias undulatus*). Gen Comp Endocrinol (1996) 104(2):231–42. 10.1006/gcen.1996.0166 8930614

[196] Gaildrat P Falcón J Melatonin receptors in the pituitary of a teleost fish: mRNA expression, 2-[125I]iodomelatonin binding and cyclic AMP response. Neuroendocrinology (2000) 72(1):57–66. 10.1159/000054571 10940739

[197] Falcón J Besseau L Fazzari D Attia J Gaildrat P Beauchaud M Melatonin modulates secretion of growth hormone and prolactin by trout pituitary glands and cells in culture. Endocrinology (2003) 144(10):4648–58. 10.1210/en.2003-0707 12960030

[198] Herrero MJ Lepesant JM Daily and seasonal expression of clock genes in the pituitary of the European sea bass (*Dicentrarchus labrax*). Gen Comp Endocrinol (2014) 208:30–8. 10.1016/j.ygcen.2014.08.002 25148807

[199] Kawabata-Sakata Y Nishiike Y Fleming T Kikuchi Y Okubo K Androgen-dependent sexual dimorphism in pituitary tryptophan hydroxylase expression: relevance to sex differences in pituitary hormones. Proc Biol Sci (2020) 287(1928):20200713. 10.1098/rspb.2020.0713 32517612 PMC7341908

[200] Confente F Rendón MC Besseau L Falcón J Muñoz-Cueto JA Melatonin receptors in a pleuronectiform species, Solea senegalensis: Cloning, tissue expression, day-night and seasonal variations. Gen Comp Endocrinol (2010) 167(2):202–14. 10.1016/j.ygcen.2010.03.006 20227412

[201] Ikegami T Azuma K Nakamura M Suzuki N Hattori A Ando H Diurnal expressions of four subtypes of melatonin receptor genes in the optic tectum and retina of goldfish. Comp Biochem Physiol - A Mol Integr Physiol (2009) 152(2):219–24. 10.1016/j.cbpa.2008.09.030 18930834

[202] Shi Q Ando H Coon SL Sato S Ban M Urano A Embryonic and post-embryonic expression of arylalkylamine N-acetyltransferase and melatonin receptor genes in the eye and brain of chum salmon (*Oncorhynchus keta*). Gen Comp Endocrinol (2004) 136(3):311–21. 10.1016/j.ygcen.2004.01.004 15081830

[203] Sauzet S Besseau L Herrera Perez P Covès D Chatain B Peyric E Cloning and retinal expression of melatonin receptors in the European sea bass, Dicentrarchus labrax. Gen Comp Endocrinol (2008) 157(2):186–95. 10.1016/j.ygcen.2008.04.008 18555069

[204] Nakane Y Yoshimura T Photoperiodic Regulation of Reproduction in Vertebrates. Annu Rev Anim Biosci (2019) 7:173–94. 10.1146/annurev-animal-020518-115216 30332291

[205] Nakane Y Ikegami K Iigo M Ono H Takeda K Takahashi D The saccus vasculosus of fish is a sensor of seasonal changes in day length. Nat Commun (2013) 4(1):2108. 10.1038/ncomms3108 23820554

[206] Irachi S Hall DJ Fleming MS Maugars G Björnsson BT Dufour S Photoperiodic regulation of pituitary thyroid-stimulating hormone and brain deiodinase in Atlantic salmon. Mol Cell Endocrinol (2020) 519:111056. 10.1016/j.mce.2020.111056 33069856

[207] Altner H Zimmermann H The Saccus Vasculosus. In: Bourne GH, editor. Structure and Physiology. New York and London: Academic Press (1972). p. 293–328. 10.1016/b978-0-12-119285-3.50012-x

[208] Wulliman FM Rupp B Reichert H Neuroanatomy of the Zebrafish Brain: A Topological Atlas. Basel, Boston, Berlin: Birkhäuser Verlag (2012). 10.1007/978-3-0348-8979-7

[209] Maugars G Dufour S Cohen-Tannoudji JL Quérat B Multiple thyrotropin b-subunit and thyrotropin receptor-related genes arose during vertebrate evolution. Robinson-Rechavi M, ed. PLoS One (2014) 9(11):e111361. 10.1371/journal.pone.0111361 25386660 PMC4227674

[210] Fleming MS Maugars G Lafont AG Rancon J Fontaine R Nourizadeh-Lillabadi R Functional divergence of thyrotropin beta-subunit paralogs gives new insights into salmon smoltification metamorphosis. Sci Rep (2019) 9(1):4561. 10.1038/s41598-019-40019-5 30872608 PMC6418267

[211] Gern WA Owens DW Ralph CL Plasma melatonin in the trout: Day-night change demonstrated by radioimmunoassay. Gen Comp Endocrinol (1978) 34(4):453–8. 10.1016/0016-6480(78)90286-1 648874

[212] Fontaine R Ager-Wick E Hodne K Weltzien F-A Plasticity of Lh cells caused by cell proliferation and recruitment of existing cells. J Endocrinol (2019) 240(2):361–77. 10.1530/JOE-18-0412 30594119

[213] Falcón J Migaud H Muñoz-Cueto JA Carrillo M Current knowledge on the melatonin system in teleost fish. Gen Comp Endocrinol (2010) 165(3):469–82. 10.1016/j.ygcen.2009.04.026 19409900

